# Comparative Genomics of Borderline Oxacillin-Resistant Staphylococcus aureus Detected during a Pseudo-outbreak of Methicillin-Resistant S. aureus in a Neonatal Intensive Care Unit

**DOI:** 10.1128/mbio.03196-21

**Published:** 2022-01-18

**Authors:** Sanjam S. Sawhney, Eric M. Ransom, Meghan A. Wallace, Patrick J. Reich, Gautam Dantas, Carey-Ann D. Burnham

**Affiliations:** a The Edison Family Center for Genome Sciences and Systems Biology, Washington University School of Medicine, St. Louis, Missouri, USA; b Department of Pathology and Immunology, Washington University School of Medicine, St. Louis, Missouri, USA; c Department of Pediatrics, Washington University School of Medicine, St. Louis, Missouri, USA; d Department of Molecular Microbiology, Washington University School of Medicine, St. Louis, Missouri, USA; e Department of Biomedical Engineering, Washington University in St. Louis, St. Louis, Missouri, USA; Brigham and Women's Hospital

**Keywords:** oxacillin, BORSA, MRSA, WGS, NGS, surveillance cultures, chromogenic agars, susceptibility testing, random forest classifier, *Staphylococcus aureus*, chromogenic media, *gdpP*, surveillance studies

## Abstract

Active surveillance for methicillin-resistant Staphylococcus aureus (MRSA) is a component of our neonatal intensive care unit (NICU) infection prevention efforts. Recent atypical trends prompted review of 42 suspected MRSA isolates. Species identification was confirmed by matrix-assisted laser desorption ionization–time of flight mass spectrometry (MALDI-TOF MS), and methicillin resistance was reevaluated by PBP2a lateral flow assay, cefoxitin/oxacillin susceptibility testing, *mecA* and *mecC* PCR, and six commercially available MRSA detection agars. All isolates were confirmed S. aureus, but only eight were MRSA (cefoxitin resistant, PBP2a positive, *mecA* positive, growth on all MRSA screening agars). One MRSA isolate was cefoxitin susceptible but PBP2a and *mecA* positive, and the remaining 33 were cefoxitin susceptible, PBP2a negative, and *mecA* negative; interestingly, these isolates grew inconsistently across MRSA screening agars and had susceptibility profiles consistent with that of borderline oxacillin-resistant S. aureus (BORSA). Comparative genomic analyses found these BORSA isolates to be phylogenetically diverse and not representative of clonal expansion or shared gene content, though clones of two NICU strains were infrequently observed over 8 months. We identified 6 features—substitutions and truncations in PBP2, PBP4, and GdpP and beta-lactamase hyperproduction—that were used to generate a random forest classifier to distinguish BORSA from methicillin-susceptible S. aureus (MSSA) in our cohort. Our model demonstrated a robust ability to predict the BORSA phenotype among isolates collected across two continents (validation area under the curve [AUC], 0.902). Taking these findings together, we observed an unexpected prevalence of BORSA in our NICU, BORSA misclassification by existing MRSA screening methods, and markers that are together discriminatory for BORSA and MSSA within our cohort. This work has implications for epidemiological reporting of MRSA rates for centers using different screening methods.

## INTRODUCTION

Methicillin-resistant Staphylococcus aureus (MRSA) is an important cause of morbidity and mortality in both health care and community settings (1). The Centers for Disease Control and Prevention (CDC) defines MRSA as S. aureus resistant to methicillin, cefoxitin, or oxacillin by standard susceptibility testing methods or by the detection of laboratory markers of methicillin resistance (2). Methicillin resistance is most often associated with the penicillin-binding protein 2a (PBP2a), encoded by *mecA*. The homolog *mecC* can also confer methicillin resistance but is far less common in the United States (3).

Resistance to beta-lactam antibiotics has been observed in some *mecA-* or *mecC*-negative S. aureus isolates; in these cases, treatment with beta-lactam agents can exacerbate disease burden and result in poorer prognoses (4, 5). Early studies from the 1980s attributed this low-level oxacillin and/or methicillin resistance to hyperproduction of beta-lactamase (6), with further work revealing that these beta-lactamase hyperproducers (BHP), termed borderline oxacillin-resistant S. aureus (BORSA), regain susceptibility upon introduction of a beta-lactamase inhibitor (7, 8). However, beta-lactamase hyperproduction alone was found to be insufficient in some isolates for borderline oxacillin resistance (9, 10), and *mecA*- and *mecC*-negative, non-BHP isolates with borderline resistance were also recovered (11–13).

A second hypothesis for borderline oxacillin resistance points to “modified” PBP proteins with lowered drug reactivity and elevated PBP4 levels (14). These isolates were found to have multiple unlinked point mutations initially in *pbp2* (11, 15, 16) and later in *pbp1*, *pbp3*, and *pbp4* (12, 13, 17). Originally termed modified PBP S. aureus (MODSA) strains (14), these isolates are increasingly also referred to as BORSA under a mechanism-agnostic nomenclature (16, 18), as is reflected here. Recently, alternate pathways involving GdpP have been described in relation to borderline oxacillin resistance (17, 19, 20), complicating our understanding of the phenotype and necessitating further investigation.

Although these *mecA*-negative borderline oxacillin-resistant isolates are considered MRSA by the CDC’s MRSA definition, there is remarkably no consensus definition for BORSA. The prevalence of BORSA reported in the literature is from <1% to 12.5% of S. aureus isolates (21), which may be partially driven by local microbial epidemiology and the MRSA surveillance screening employed within hospital infection prevention practices.

In early 2020, an investigation led by the infection prevention team at Saint Louis Children’s Hospital identified instances of irregular positivity from MRSA screening cultures collected from the anterior nares of patients in the neonatal intensive care unit (NICU) in 2019. These instances included scenarios such as a patient with a single positive culture flanked by weekly negative cultures, a patient with an initial positive culture at birth followed by only negative cultures, and patients with sporadic positive cultures spanning an extended time frame. The microbiology laboratory at Barnes-Jewish Hospital characterized these 42 suspicious isolates, along with 60 comparator isolates recovered from positive blood cultures, by antimicrobial susceptibility testing, genotypic and phenotypic MRSA surrogate marker detection (e.g., *mecA* and PBP2a, respectively), and whole-genome sequencing (WGS). Our objective was to profile the genomic relatedness of these isolates for outbreak assessment, generate a computational model to identify genomic correlates of the BORSA phenotype, and evaluate the performance of MRSA screening agars to detect MRSA and BORSA.

## RESULTS

### Characterization of isolates from the NICU.

**FIG 1:**
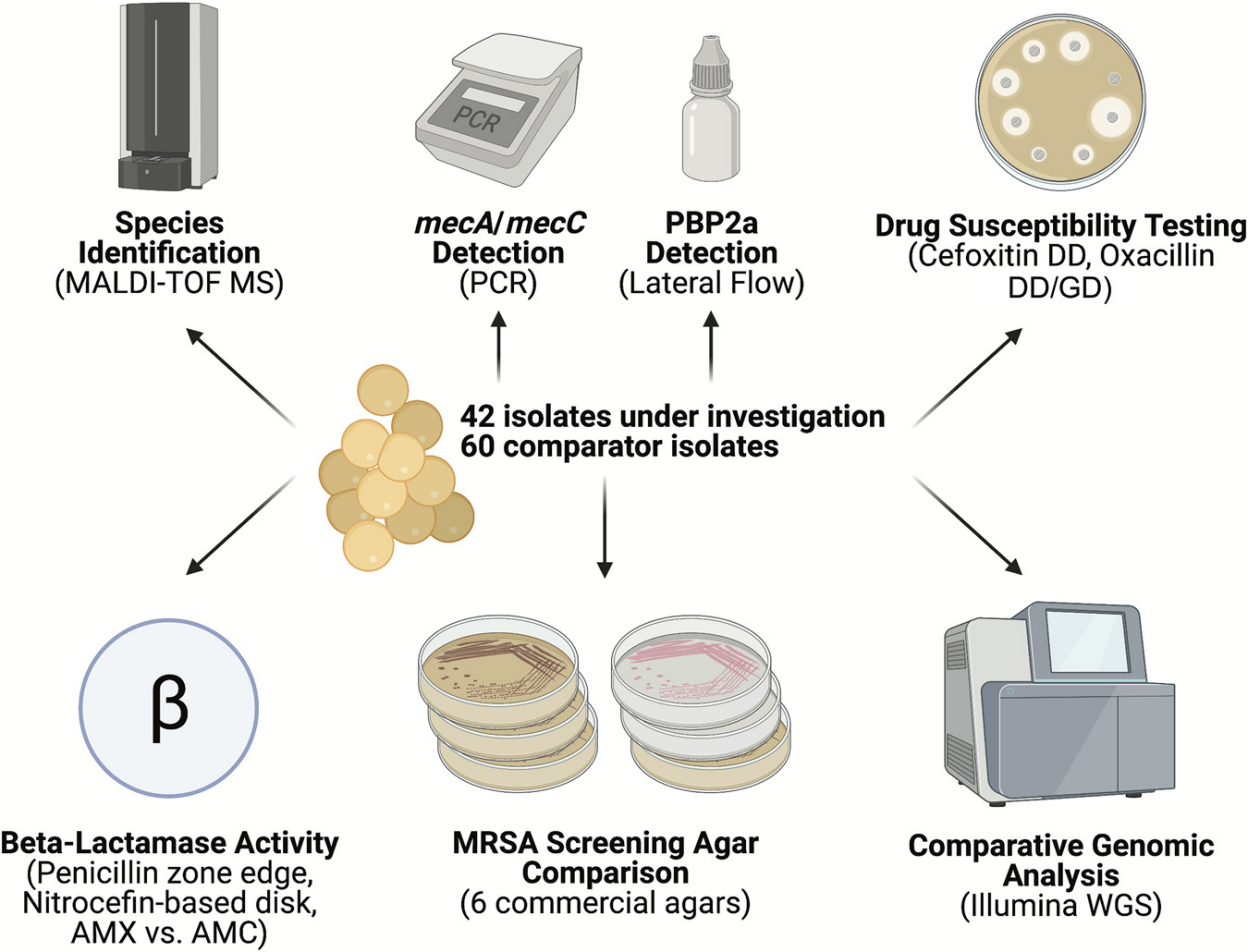
Experimental schematic. Overview of diagnostic methods employed to interrogate clinical S. aureus isolates for oxacillin resistance. (The image was made with BioRender.) AMX, amoxicillin; AMC, amoxicillin-clavulanic acid; DD, disk diffusion; GD, gradient diffusion.

Forty-two isolates were characterized as part of a NICU MRSA screening culture investigation (Fig. 1). These isolates were originally reported as MRSA based on growth characteristics on Spectra MRSA screening agar. This study began by confirming that the 42 isolates were S. aureus using matrix-assisted laser desorption ionization–time of flight mass spectrometry (MALDI-TOF MS). To confirm methicillin resistance, isolates were screened for PBP2a and the PBP2a-encoding gene, *mecA*, using a rapid qualitative immunochromatographic assay and PCR, respectively. Interestingly, only 9 isolates tested positive for PBP2a (Table 1). In support, *mecA* was detected in only the 9 PBP2a-positive isolates and was not detected in the PBP2a-negative isolates (Table 1). Of note, one PBP2a/*mecA*-positive isolate was from a culture with two S. aureus colony morphologies (see Table S1 in the supplemental material). An in-house PCR for the *mecA* homolog *mecC* was also performed (22, 23), with no isolates returning a positive result (Table S1). Ultimately, 33 isolates remained in question.

**TABLE 1:** Phenotypic and genotypic characterization of index isolates, including phenotypic susceptibility testing and evaluation of chromogenic culture medium^*a*^

Isolate	Class	Result of molecular testing		Result of susceptibility testing			Result with MRSA screening agar(s):			
		PBP2a	*mecA* PCR	FOX DD, MH	OXA DD, MH	OXA GD, 2% NaCl MH	Spectra MRSA	HardyCHROM MRSA	Nonchromogenic MRSA screen agar	Other 3 agars^*b*^
301	BORSA	–	–	S	R	R	+++	+++	+++	–
302	BORSA	–	–	S	I	S	+++	+++	–	–
303	MRSA	P	Det	R	R	R	+++	+++	+++	+++
304	MSSA	–	–	S	S	S	+	–	–	–
305	BORSA	–	–	S	R	R	++	+++	–	–
306	BORSA	–	–	S	S	R	++	+++	–	–
307	BORSA	–	–	S	S	R	++	–	–	–
308	MRSA	P	Det	R	R	R	+++	+++	+++	+++
309	MRSA	P	Det	R	R	R	+++	+++	+++	+++
310	BORSA/MSSA^*c*^	–	–	S	S	R	++	–	–	–
311	MSSA	–	–	S	S	S	++	++	–	–
312	MSSA	–	–	S	S	S	++	–	–	–
314	MSSA	–	–	S	S	S	+++	–	–	–
315	BORSA	–	–	S	S	R	++	–	–	–
316	BORSA	–	–	S	S	R	+	–	–	–
318	BORSA	–	–	S	R	R	+	–	–	–
320	MRSA	P	Det	R	R	R	+++	+++	+++	+++
321	BORSA	–	–	S	R	R	+++	+++	+++	–
322	MRSA	P	Det	R	R	R	+++	+++	+++	+++
323	BORSA	–	–	S	S	R	+++	++	+++	–
324	MRSA	P	Det	R	R	R	+++	+++	+++	+++
325	MSSA	–	–	S	S	S	+	–	–	–
327	BORSA	–	–	S	S	R	+++	–	–	–
328	BORSA	–	–	S	I	R	++	–	–	–
329	MRSA	P	Det	R	R	R	+++	++	+++	++
330	MRSA	P	Det	R	R	R	+++	+	+++	+++
331	MSSA	–	–	S	S	S	++	–	–	–
332	BORSA	–	–	S	S	R	+	–	–	–
333	MSSA	–	–	S	S	S	++	–	–	–
334	BORSA	–	–	S	S	R	+	–	–	–
335	MRSA	P	Det	S	I	S	++	–	+++	–
336	BORSA	–	–	S	R	R	+	++	++	–
337	BORSA	–	–	S	S	R	+++	+	++	–
338	BORSA	–	–	S	S	R	+	–	++	–
339	BORSA	–	–	S	S	R	+++	+++	++	–
340	BORSA	–	–	S	S	R	+++	++	++	–
341	BORSA	–	–	S	S	R	++	++	++	–
342	BORSA	–	–	S	S	R	++	++	++	–
343	MSSA	–	–	S	S	S	–	++	–	–
344	BORSA	–	–	R	R	R	+++	+++	++	–
345	BORSA	–	–	S	S	R	+++	–	–	–
346	BORSA	–	–	R	R	R	+++	+++	++	–

a Abbreviations: FOX, cefoxitin; OXA, oxacillin; DD, disk diffusion; GD, gradient diffusion; MH, Mueller-Hinton agar; P, positive; –, negative, not detected, or no growth; Det, detected; S, susceptible; I, intermediate; R, resistant. Plus signs indicate growth abundance, as follows: rare growth (+), a few cells (++), or growth equal to that of the control strain (+++).

b The agars MRSA*Select* II, BBL CHROMagar MRSA II, and chromID MRSA performed identically.

c This isolate shared characteristics consistent with BORSA and MSSA (see Discussion for details).

10.1128/mbio.03196-21.7 TABLE S1 Compiled data on isolates under investigation and comparator blood isolates. Download Table S1, DOCX file, 0.05 MB. Copyright © 2022 Sawhney et al. 2022 Sawhney et al. https://creativecommons.org/licenses/by/4.0/ This content is distributed under the terms of the Creative Commons Attribution 4.0 International license.

An additional 60 S. aureus isolates from blood cultures of 60 patients were also included in this study to serve as comparators to contextualize the NICU isolates. These isolates include 50 consecutive S. aureus isolates recovered from blood cultures at the Barnes Jewish microbiology laboratory (regardless of age or patient location) and all non-MRSA isolates recovered from blood cultures of NICU patients in 2019 (*n* = 10). All 60 isolates were confirmed to be S. aureus by MALDI-TOF MS, negative for PBP2a, and cefoxitin susceptible, with *mecA*/*mecC* not detected by PCR (Table 2 and Table S1), initially indicating methicillin-susceptible S. aureus (MSSA) status. These isolates are here referred to as the comparator blood isolates.

**TABLE 2:** Phenotypic and genotypic characterization of comparator blood isolates, including phenotypic susceptibility testing and evaluation of chromogenic culture medium^*a*^

Isolate	Class	Result of molecular testing		Result of susceptibility testing			Result with MRSA screening agar(s):			
		PBP2a	*mecA* PCR	FOX DD, MH	OXA DD, MH	OXA GD, 2% NaCl MH	Spectra MRSA	HardyCHROM MRSA	Nonchromogenic MRSA screen	Other 3 agars^*b*^
1	MSSA	–	–	S	S	S	–	–	–	–
2	MSSA	–	–	S	S	S	–	–	–	–
3	MSSA	–	–	S	S	S	–	–	–	–
4	MSSA	–	–	S	S	S	–	–	–	–
5	MSSA	–	–	S	S	S	–	–	–	–
6	MSSA	–	–	S	S	S	–	–	–	–
7	MSSA	–	–	S	S	S	–	–	–	–
8	MSSA	–	–	S	S	S	–	–	–	–
9	MSSA	–	–	S	S	S	–	–	–	–
10	BORSA	–	–	S	S	R	–	–	–	–
11	MSSA	–	–	S	S	S	+	–	–	–
12	BORSA	–	–	S	S	R	–	++	–	–
13	MSSA	–	–	S	S	S	–	–	–	–
14	MSSA	–	–	S	S	S	–	–	–	–
15	MSSA	–	–	S	S	S	–	–	–	–
16	MSSA	–	–	S	S	S	–	–	–	–
17	MSSA	–	–	S	S	S	–	–	–	–
18	MSSA	–	–	S	S	S	–	–	–	–
19	MSSA	–	–	S	S	S	–	–	–	–
20	BORSA	–	–	S	S	R	–	–	–	–
21	MSSA	–	–	S	S	S	–	–	–	–
22	MSSA	–	–	S	S	S	–	++	–	–
23	BORSA	–	–	S	S	R	–	+	–	–
24	MSSA	–	–	S	S	S	–	–	–	–
25	MSSA	–	–	S	S	S	–	–	–	–
26	BORSA	–	–	S	S	R	++	–	+	–
27	MSSA	–	–	S	S	S	–	–	–	–
28	MSSA	–	–	S	S	S	–	–	–	–
29	BORSA	–	–	S	S	R	–	–	–	–
30	MSSA	–	–	S	S	S	–	–	–	–
31	MSSA	–	–	S	S	S	–	–	–	–
32	MSSA	–	–	S	S	S	–	–	–	–
33	MSSA	–	–	S	S	S	–	–	–	–
34	BORSA	–	–	S	S	R	–	–	–	–
35	MSSA	–	–	S	S	S	–	–	–	–
36	MSSA	–	–	S	S	S	–	–	–	–
37	BORSA	–	–	S	S	R	–	–	–	–
38	MSSA	–	–	S	S	S	–	–	–	–
39	MSSA	–	–	S	S	S	–	–	–	–
40	MSSA	–	–	S	S	S	–	–	–	–
41	MSSA	–	–	S	S	S	–	–	–	–
42	MSSA	–	–	S	S	S	–	–	–	–
43	MSSA	–	–	S	S	S	–	–	–	–
44	MSSA	–	–	S	S	S	–	–	–	–
45	MSSA	–	–	S	S	S	–	–	–	–
46	MSSA	–	–	S	S	S	–	–	–	–
47	MSSA	–	–	S	S	S	–	–	–	–
48	MSSA	–	–	S	S	S	–	–	–	–
49	BORSA	–	–	S	S	R	–	–	–	–
50	MSSA	–	–	S	S	S	–	–	–	–
53	MSSA	–	–	S	S	S	–	–	–	–
54	MSSA	–	–	S	S	S	+	–	–	–
55	MSSA	–	–	S	S	S	–	–	–	–
56	MSSA	–	–	S	S	S	–	–	–	–
57	MSSA	–	–	S	S	S	–	–	–	–
58	MSSA	–	–	S	S	S	–	–	–	–
59	MSSA	–	–	S	S	S	++	–	–	–
60	MSSA	–	–	S	S	S	–	–	–	–
61	MSSA	–	–	S	S	S	–	–	–	–
62	MSSA	–	–	S	S	S	++	–	–	–

a Abbreviations: FOX, cefoxitin; OXA, oxacillin; DD, disk diffusion; GD, gradient diffusion; MH, Mueller-Hinton agar; P, positive; –, negative, not detected, or no growth; Det, detected; S, susceptible; R, resistant. Plus signs indicate growth abundance, as follows: rare growth (+), a few cells (++), or growth equal to that of the control strain (+++).

b The agars MRSA*Select* II, BBL CHROMagar MRSA II, and chromID MRSA performed identically.

### Spectra MRSA screening agar.

**FIG 2:**
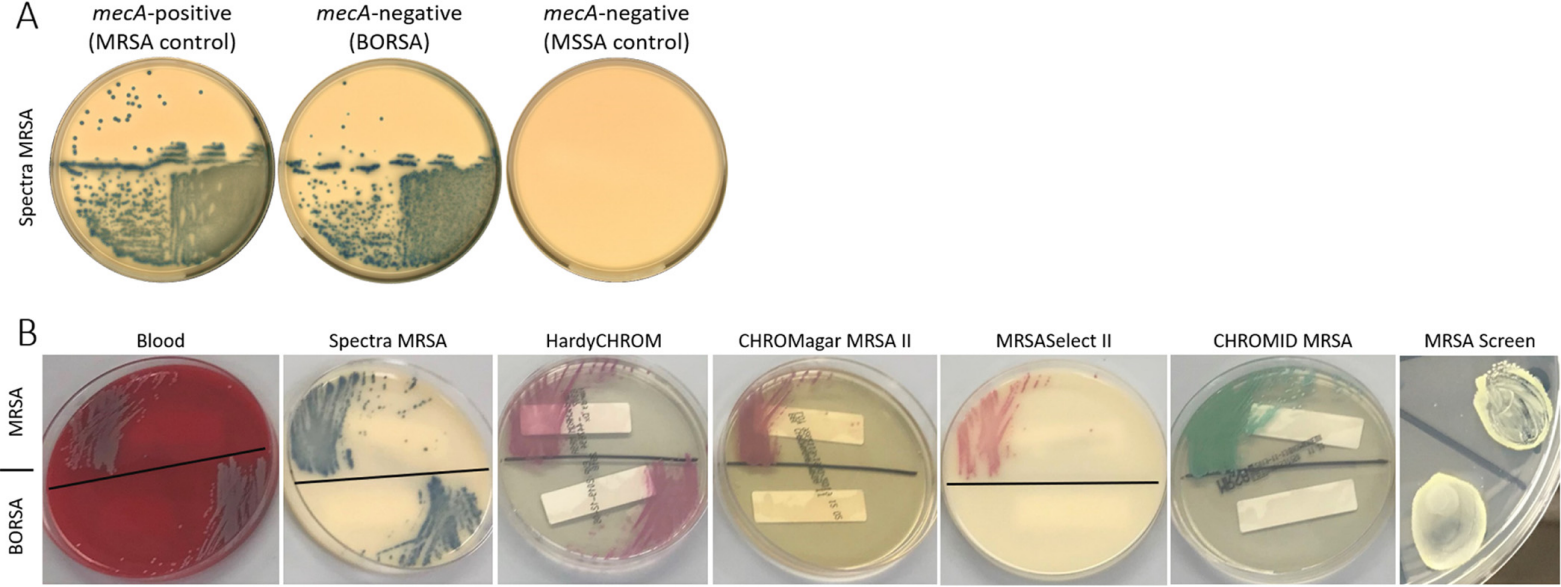
Comparison of levels of growth on MRSA screening agars. (A) Representative images of MRSA, BORSA, and MSSA isolates grown on Spectra MRSA screening agar. (B) Representative images of growth and pigmentation from different screening agars using a MRSA isolate (top) and BORSA isolate (bottom) grown on the same agar plate.

Potential explanations for recovering non-MRSA isolates from the MRSA screening agar are faulty media and inaccuracies in interpreting the growth on the culture media. To determine whether these factors contributed, the 42 isolates (including the 9 confirmed MRSA isolates) were retested on Spectra MRSA screening agar, with representative images shown in Fig. 2A. Interestingly, all but one isolate exhibited growth and demonstrated the expected denim-blue pigmentation for MRSA. The growth patterns were somewhat varied in terms of colony size and abundance (as expected based on further resistance mechanism characterization [see below]). In general, the confirmed MRSA isolates had the most robust growth, as expected. However, the majority of the remaining isolates exhibited reduced growth (e.g., smaller colony sizes and/or reduced numbers of CFU compared to those of MRSA control strains) (Table 1). The discrepancies between the Spectra MRSA screening agar and the findings from the PBP2a and *mecA* PCR testing were surprising; they were in stark contrast to the findings for 60 comparator blood isolates, where 55 isolates had no growth, 3 had robust growth, and 2 had rare growth (Table 2).

### Cefoxitin and oxacillin susceptibility testing.

According to the Clinical and Laboratory Standards Institute (CLSI), resistance to cefoxitin via disk diffusion is a surrogate marker to predict *mecA*-mediated methicillin resistance (MRSA). To determine cefoxitin resistance, the 42 isolates were tested using the disk diffusion method as described by the CLSI. Eight of the 9 MRSA isolates (PBP2a- and *mecA*-positive isolates) were resistant (Table 1). One MRSA isolate was susceptible to cefoxitin (zone size = 25 mm). Two more isolates tested resistant near the breakpoint (both, 19 mm) but were PBP2a and *mecA* negative. None of the 60 comparator blood isolates tested cefoxitin resistant.

Susceptibility testing was also performed for oxacillin resistance. First, isolates were tested by the oxacillin disk diffusion method and interpreted using archived 2007 CLSI breakpoints (24). These historical oxacillin breakpoints were archived when the focus shifted specifically to detection of *mecA*-mediated beta-lactam resistance. Of the 42 isolates, 15 were resistant, including the 8 confirmed MRSA isolates (Table 1). The lone cefoxitin-susceptible MRSA isolate was oxacillin intermediate. Two additional isolates were also oxacillin intermediate. None of the 60 comparator blood isolates tested oxacillin resistant or intermediate by disk diffusion. Following this, isolates were tested for oxacillin resistance using a previously reported gradient diffusion method that induces resistance by plating cells on 2% NaCl screening agar (25–27). Thirty-three of the 42 isolates were found to be resistant (Table 1). Nine of the 60 comparator blood isolates also tested oxacillin resistant (Table 2). For subsequent analyses, BORSA isolates were defined using the following criteria: *mecA*/PBP2a-negative S. aureus and (i) resistant or intermediate to oxacillin by disk diffusion (zone size ≤ 12 mm) or (ii) resistant to oxacillin by gradient diffusion using inducible 2% NaCl agar (consistent MIC ≥ 4 μg/mL tested in triplicate). This definition captured 24 of the 33 non-MRSA investigated isolates, and 9 comparator isolates, as BORSA; the 9 comparator BORSA isolates were investigated, and 51 comparator isolates were designated MSSA. The nine BORSA isolates from the comparator collection were from 9 patients with an age range of 7 to 77 years (median age of 49 years) who were receiving care at three different hospitals, suggesting that the isolates with this phenotype were not restricted to the neonatal ICU. Further investigation found no epidemiological commonalities associated with these nine isolates from the comparator collection.

Beta-lactamase hyperproduction is a non-*mecA* resistance mechanism that may contribute to the above-described BORSA phenotype (6–8). To detect beta-lactamase production in these S. aureus isolates, two CLSI-recommended tests were performed: the disk diffusion penicillin zone edge test and a nitrocefin-based test. The disk diffusion method demonstrated that 38 investigated isolates were beta-lactamase positive (Table S1). These findings reflected the nitrocefin test results, except for isolate 308, which had a negative Cefinase test. Of the 60 comparator blood isolates, 44 were positive by both methods, 15 were negative by both methods, and isolate 56 was Cefinase positive and zone edge test negative (Table S1).

Hyperproduction of beta-lactamase has previously been distinguished as a resistance mechanism in BORSA isolates when the addition of a beta-lactamase inhibitor lowers the MIC of the parent drug by at least 2 dilutions when tested in combination with the beta-lactamase inhibitor (13). All isolates were tested against amoxicillin and amoxicillin-clavulanate acid using the established gradient diffusion method. Six of the 33 isolates defined as BORSA exhibited a 4-fold difference in lactamase inhibitor effect (Table S1), while just 6 isolates of the 60 isolates defined as MSSA also exhibited a 4-fold difference (Table S1); taken together, the difference in lactamase inhibitor effect between BORSA and MSSA isolates was significant (*P* = 0.0030, Mann-Whitney test) (Fig. S1).

10.1128/mbio.03196-21.1 FIG S1 Beta-lactamase inhibitor effect. Log_2_ fold change in MICs between amoxicillin and amoxicillin-clavulanic acid. Lines represent standard deviations, and significance is measured by the Mann-Whitney U test. Download FIG S1, PDF file, 0.02 MB. Copyright © 2022 Sawhney et al. 2022 Sawhney et al. https://creativecommons.org/licenses/by/4.0/ This content is distributed under the terms of the Creative Commons Attribution 4.0 International license.

### MRSA screening agar comparison.

To determine how these S. aureus isolates with differing cefoxitin and oxacillin resistance profiles performed on other commercially available MRSA screening media, 5 additional MRSA screening agars were evaluated: MRSA*Select* II, BBL CHROMagar MRSA II, chromID MRSA, nonchromogenic MRSA screen agar, and HardyCHROM MRSA. The 8 cefoxitin-resistant MRSA isolates grew on all agars and were the only isolates to grow on MRSA*Select* II, BBL CHROMagar MRSA II, and chromID MRSA. The nonchromogenic MRSA screen agar grew an additional 13 isolates (12 BORSA isolates and the lone cefoxitin-susceptible MRSA isolate), while the HardyCHROM MRSA agar grew an additional 16 isolates (14 BORSA, 2 MSSA isolates) (Table 1). For the comparator blood isolates, none of the 60 isolates grew on MRSA*Select* II, BBL CHROMagar MRSA II, and chromID MRSA agars; however, one BORSA isolate grew on the nonchromogenic MRSA screen agar, and 3 isolates (2 BORSA isolates, 1 MSSA isolate) grew on the HardyCHROM MRSA agar (Table 2). Taken together, these findings suggested differing selective properties across commercially available agars, resulting in different isolates being flagged as MRSA, irrespective of PBP2a and *mecA* presence. Representative images are shown in Fig. 2B.

To evaluate the analytical performance of the different MRSA screening agars to detect MRSA and BORSA, sensitivity and specificity were calculated using all 102 isolates. As shown in Table 3, sensitivities and specificities varied by the agar used and its ability to detect MRSA and BORSA. Briefly, for *mecA*-mediated MRSA detection, the BBL CHROMagar MRSA II, MRSA*Select* II, and chromID MRSA agars demonstrated 89% sensitivity and 100% specificity. The nonchromogenic MRSA screen agar and Spectra MRSA agar had 100% sensitivity and specificities of 86% and 60%, respectively. For the detection of *mecA*-positive isolates and BORSA isolates using criterion i, the BBL CHROMagar MRSA II, MRSA*Select* II, and chromID MRSA agars had 50% sensitivity and 100% specificity. Spectra MRSA agar exhibited 100% sensitivity and 65% specificity. For detection of *mecA*-positive isolates and BORSA isolates using criteria ii, the BBL CHROMagar MRSA II, MRSA*Select* II, and chromID MRSA agars had 20% sensitivity and 100% specificity. The nonchromogenic MRSA screen agar and Spectra MRSA agar had, respectively, sensitivities of 54% and 76% and specificities of 100% and 83%. While no screening agar had perfect analytical performance characteristics, laboratories may opt to use a specific agar depending on the desire to detect MRSA and BORSA isolates or to focus detection specifically on *mecA*-positive strains.

**TABLE 3:** Sensitivities and specificities of commercially available MRSA screening agars

Agar	*mecA*-mediated detection only		With BORSA criterion i^*a*^		With BORSA criterion ii^*b*^	
	Sensitivity	Specificity	Sensitivity	Specificity	Sensitivity	Specificity
BBL CHROMagar MRSA II	0.89	1	0.5	1	0.2	1
MRSA*Select* II	0.89	1	0.5	1	0.2	1
chromID MRSA	0.89	1	0.5	1	0.2	1
Hardy MRSA screen	1	0.86	0.88	0.91	0.54	1
HardyCHROM MRSA	0.89	0.8	0.88	0.85	0.56	0.93
Spectra MRSA	1	0.6	1	0.65	0.76	0.83

a Resistant or intermediate to oxacillin by disk diffusion.

b Resistant to oxacillin by gradient diffusion using inducible 2% NaCl agar. There were 102 strains included in this evaluation.

### WGS reveals that the BORSA phenotype is not linked to the core or accessory genome.

Prior investigations into mechanisms of borderline oxacillin resistance in S. aureus isolates have been limited to amplicon sequencing of preselected genes following reported phenotypic associations. Whole-genome sequencing (WGS) of human clinical BORSA isolates is rarely performed (12, 20, 28), and only one draft assembly is presently available in the NCBI database (18). Here, we performed WGS on the 33 human clinical BORSA isolates, as well as the 9 MRSA and 60 MSSA isolates. One MSSA isolate (isolate 35) was dropped due to low coverage, resulting in 101 high-quality isolate assemblies with >99% completeness and <1% contamination (Table S2).

10.1128/mbio.03196-21.8 TABLE S2 Assembly statistics for each sequenced isolate. Download Table S2, DOCX file, 0.06 MB. Copyright © 2022 Sawhney et al. 2022 Sawhney et al. https://creativecommons.org/licenses/by/4.0/ This content is distributed under the terms of the Creative Commons Attribution 4.0 International license.

**FIG 3:**
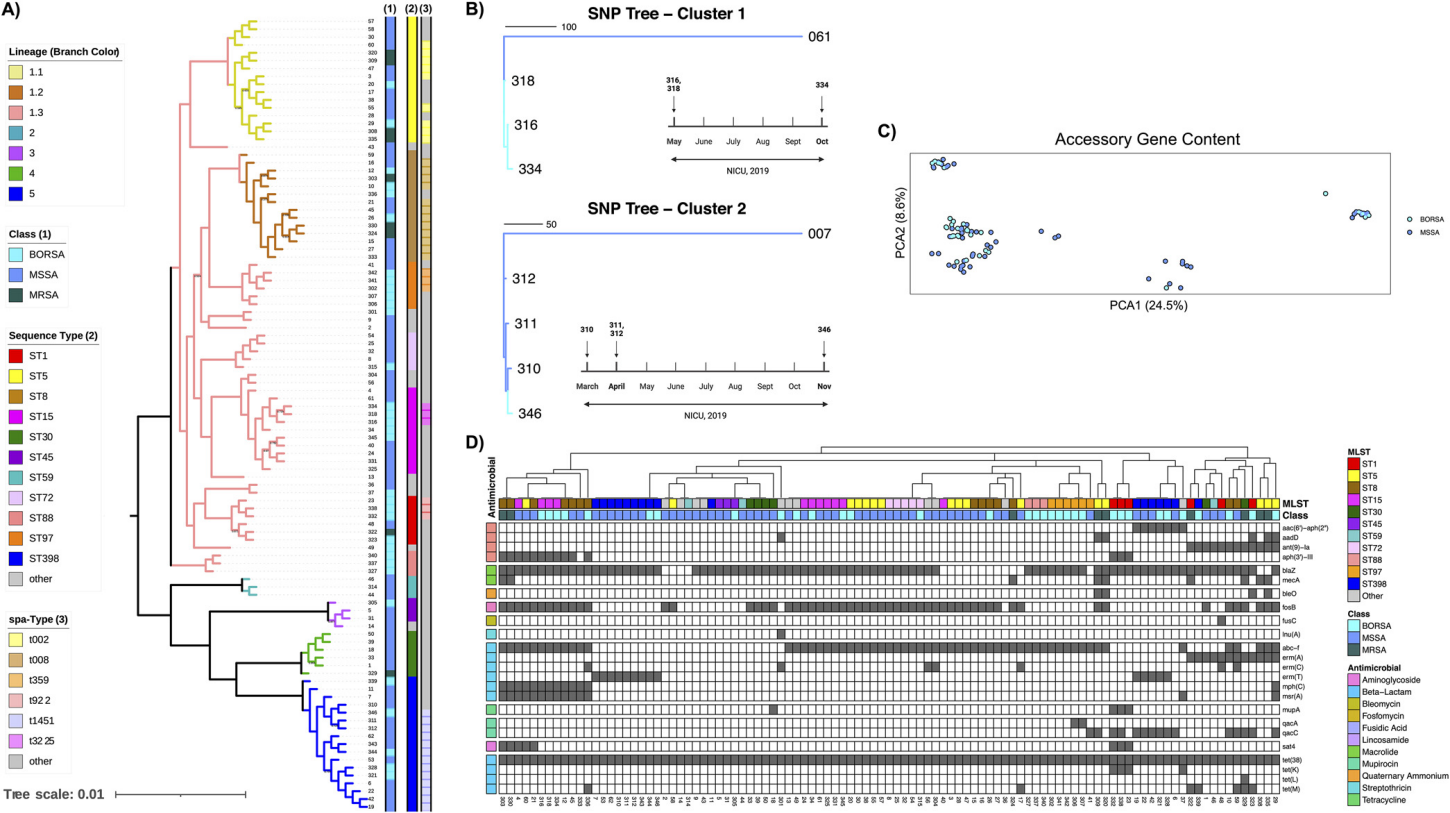
BORSA isolates do not cluster by core genome, accessory genome, or AMR content. (A) Core genome alignment of 101 S. aureus isolates. Class, MLST, and *spa* type are indicated by color strips, and lineage and clades are indicated by branch color. (B) Outgroup-rooted whole-genome SNP distance trees of clonal isolates from multiple inpatients, with timelines of collection below. Dark-blue and sky-blue branch colors represent BORSA and MSSA statuses, respectively. Cluster 1 isolates 316, 318, and 334 are 10 to 17 SNPs apart and 592 to 608 SNPs apart from their nearest phylogenetic neighbor, 061. Cluster 2 isolates 310, 311, 312, and 346 are 6 to 17 isolates apart and 394 to 400 SNPs apart from their nearest phylogenetic neighbor, 007. (C) Principal-coordinate analysis ordination of accessory genome similarity as calculated by Jaccard distance. Axis length is reflective of percent variance captured. (D) Isolates clustered by resistance gene content, with MLST and class indicated by color strips. Resistance gene rows are grouped and labeled by antimicrobial class.

To determine the population structure of BORSA within our S. aureus cohort and conduct a gene-unbiased investigation, we annotated all protein coding sequences of each isolate genome with Prokka and constructed a core genome alignment with Roary (29, 30). From this, we generated a maximum-likelihood phylogenetic tree reflecting 1,859 genes shared among >99% of isolates at >95% identity, using FastTree and iTOL (31, 32) (Fig. 3A). All major branch points indicated >90% bootstrap support values, demonstrating high confidence in the phylogenetic reconstruction. Lineages and clusters were identified with hierBAPS and annotated, depicting the evolutionary relationships of the S. aureus genomes within our cohort (33). Though five lineages were identified, more than half of the isolates within our data set were members of lineage 1. BORSA isolates represented 12 different multilocus sequence types (MLST), with those from ST398, ST15, ST97, and ST8 comprising 57.6% of the BORSA cohort. Two of the most prevalent BORSA sequence types, ST97 (*n* = 5) and ST398 (*n* = 5), have historically been contextualized as livestock-associated MRSA (34–38), though recent reports from France and the United States have described ST398 isolates of our predominating *spa* type, t1451, as human bloodstream infection (BSI)- and hospital-associated MSSA isolates (39–41), reflecting the demographics of our cohort. Moreover, a German study also found ST398 to be the third-most-common sequence type among their BORSA isolates of clinical origin (42). Yet, to the best of our knowledge, only five ST97 isolates have previously been annotated as BORSA (20, 43). Conversely, ST25, often associated with borderline oxacillin resistance in the literature (13, 16), was absent from our data set. We believe that these are the first reports of BORSA within ST27 (*n* = 1) and ST72 (*n* = 1).

Given the longitudinal collection scheme of this cohort, we assessed for both the potential of a same-strain BORSA outbreak within our NICU and the presence of S. aureus strains that may be longitudinally detected within our hospital system. For this, we employed snp-sites and snippy to identify clusters of isolates that are within 30 whole-genome single nucleotide polymorphisms (SNPs) of each other. Of the 101 analyzed isolates, we uncovered two clusters comprising 4 BORSA and 3 MSSA isolates combined from 7 patients spanning 8 months in the same NICU (Fig. 3B), highlighting existing but infrequent longitudinal detection of S. aureus strains that colonized nonoverlapping inpatients within our hospital system and providing evidence against a same-strain BORSA outbreak.

Though the core genome alignment strongly reflected genomic similarity among isolates of shared MLST and *spa* types, we strikingly did not observe strong clustering of isolates by oxacillin resistance (Fig. 3A). Moreover, the distributions of all lineages and ST groups by BORSA/MSSA status were not significantly different (*P > *0.30 and *P > *0.35, respectively; false-discovery rate [FDR]-corrected Fisher’s exact test) (Fig. S2). Through ancestral-state reconstruction of the borderline oxacillin resistance phenotype, we found independent acquisition in 17 isolates, with the remaining 16 isolates being represented among six shallow monophyletic clades of 2 to 3 isolates each (Fig. S3). Taken together, these data suggest multiple recent and sporadic acquisitions of borderline oxacillin resistance among formerly MSSA isolates, with occasional vertical transfer from an immediate ancestor.

10.1128/mbio.03196-21.2 FIG S2 Class distribution by core genome lineage and sequence type (MLST). Download FIG S2, PDF file, 0.04 MB. Copyright © 2022 Sawhney et al. 2022 Sawhney et al. https://creativecommons.org/licenses/by/4.0/ This content is distributed under the terms of the Creative Commons Attribution 4.0 International license.

10.1128/mbio.03196-21.3 FIG S3 Ancestral character estimation of borderline oxacillin resistance. Highly probable ancestral states are defined as internal nodes with a ≥0.8 likelihood of ancestral BORSA status. Shaded clades indicate BORSA isolates stemming from a recent common ancestor expected to be borderline oxacillin resistant. Download FIG S3, PDF file, 0.1 MB. Copyright © 2022 Sawhney et al. 2022 Sawhney et al. https://creativecommons.org/licenses/by/4.0/ This content is distributed under the terms of the Creative Commons Attribution 4.0 International license.

Given the lack of clustering by core genome alignment, we considered whether BORSA isolates may instead be united by similar accessory genomes. Consistently with previous reports (44–46), we observed an open pangenome architecture among our S. aureus isolates, implying sufficient depth for isolate discrimination by accessory gene content. Ordination by accessory gene content, however, did not support a distinction among isolates for borderline oxacillin resistance (Fig. 3C). A more targeted analysis surveying antimicrobial resistance (AMR) gene content validated *mecA* PCR results for MRSA isolates and found that resistance gene repertoires correlated with isolate ST. However, AMR analysis similarly found no clustering by borderline oxacillin resistance, implying that, aside from this shared phenotype, BORSA isolates have a nonuniform array of resistance repertoires (Fig. 3D). In summary, we did not observe a common BORSA signature by core genome alignment, accessory genome content, MLST, or encoded AMR profiling.

### Diverse substitutions in canonical BORSA-associated proteins.

**FIG 4:**
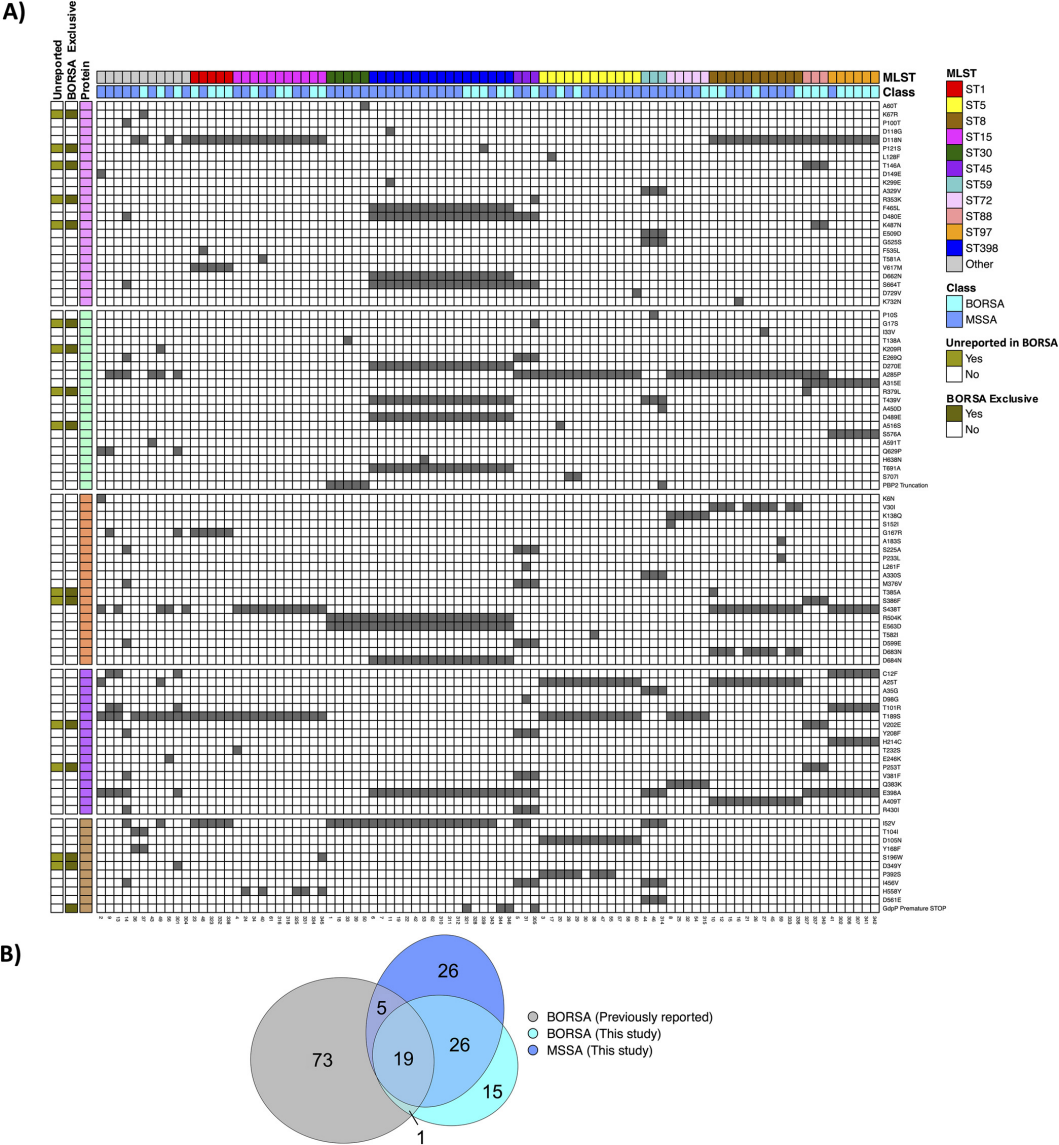
BORSA mutations by protein. (A) Consensus sequences were generated from multiple sequence alignments of PBP1 to -4 and GdpP. Amino acid mutations against these consensus sequences are visualized, with isolates clustered by MLST. For each mutation observed only in BORSA isolates within our cohort (BORSA Exclusive), the “Unreported” bar indicates whether it has previously been referenced in BORSA-related literature (11–13, 16, 17, 19, 47, 50, 51). (B) Venn diagram contrasting BORSA-linked mutations from prior reports (11–13, 16, 17, 19, 47, 50, 51), mutations present in BORSA isolates from this study, and mutations present in MSSA isolates from this study.

Oxacillin and/or methicillin resistance in *mecA-* or *mecC*-negative S. aureus isolates has historically been associated with multiple unlinked amino acid substitutions in the PBP proteins (12, 14–16, 47) and GdpP (17, 19, 20). We used Prokka to identify and extract amino acid sequences of these proteins from all BORSA and MSSA isolates to generate protein-specific amino acid alignments, from which we assembled a consensus sequence for each protein of interest and identified isolate-specific substitutions and truncations (48, 49) (Table S3). We compared our observations with mutational profiles of proteins of interest of 86 clinical and lab-grown BORSA isolates from prior reports (11–13, 16, 17, 19, 47, 50, 51). Though we similarly observed a breadth of substituted sites across the five proteins (Fig. 4A), only 37% of unique amino acid substitutions found in our cohort have been reported in published BORSA association studies (Fig. 4B). Remarkably, of these amino acid substitutions present in our isolates that were previously reported, 96% are jointly or exclusively found in our MSSA cohort as well. Even within our cohort, BORSA isolates did not definitively cluster by their substitutional profile (Fig. 4A), and a majority of mutated residues within these five proteins were found within both BORSA and MSSA isolates (Fig. 4B). Both the paucity of overlapping substitution sites across studies and the abundance of overlap between our BORSA and MSSA isolates may be attributable to the geographic distance in isolate collection sites, as most published isolates were collected in Belgium, Canada, or Scotland, while our BORSA and MSSA isolates were collected from St. Louis, MO, USA. These data indicate that even among canonical proteins of interest, BORSA isolates are characterized by a constellation of amino acid substitutions and protein truncations that are often simultaneously present in MSSA isolates.

10.1128/mbio.03196-21.9 TABLE S3 List of amino acid substitutions by BORSA isolate. Amino acid substitutions in the transglycosylase and transpeptidase domains of the PBP proteins are in italics and bold, respectively. Stop codons and deletions are red. Download Table S3, DOCX file, 0.02 MB. Copyright © 2022 Sawhney et al. 2022 Sawhney et al. https://creativecommons.org/licenses/by/4.0/ This content is distributed under the terms of the Creative Commons Attribution 4.0 International license.

### A sparse RFC identifies correlates and anticorrelates of the BORSA phenotype among isolates of varied oxacillin susceptibilities from American and Belgian cohorts.

**FIG 5:**
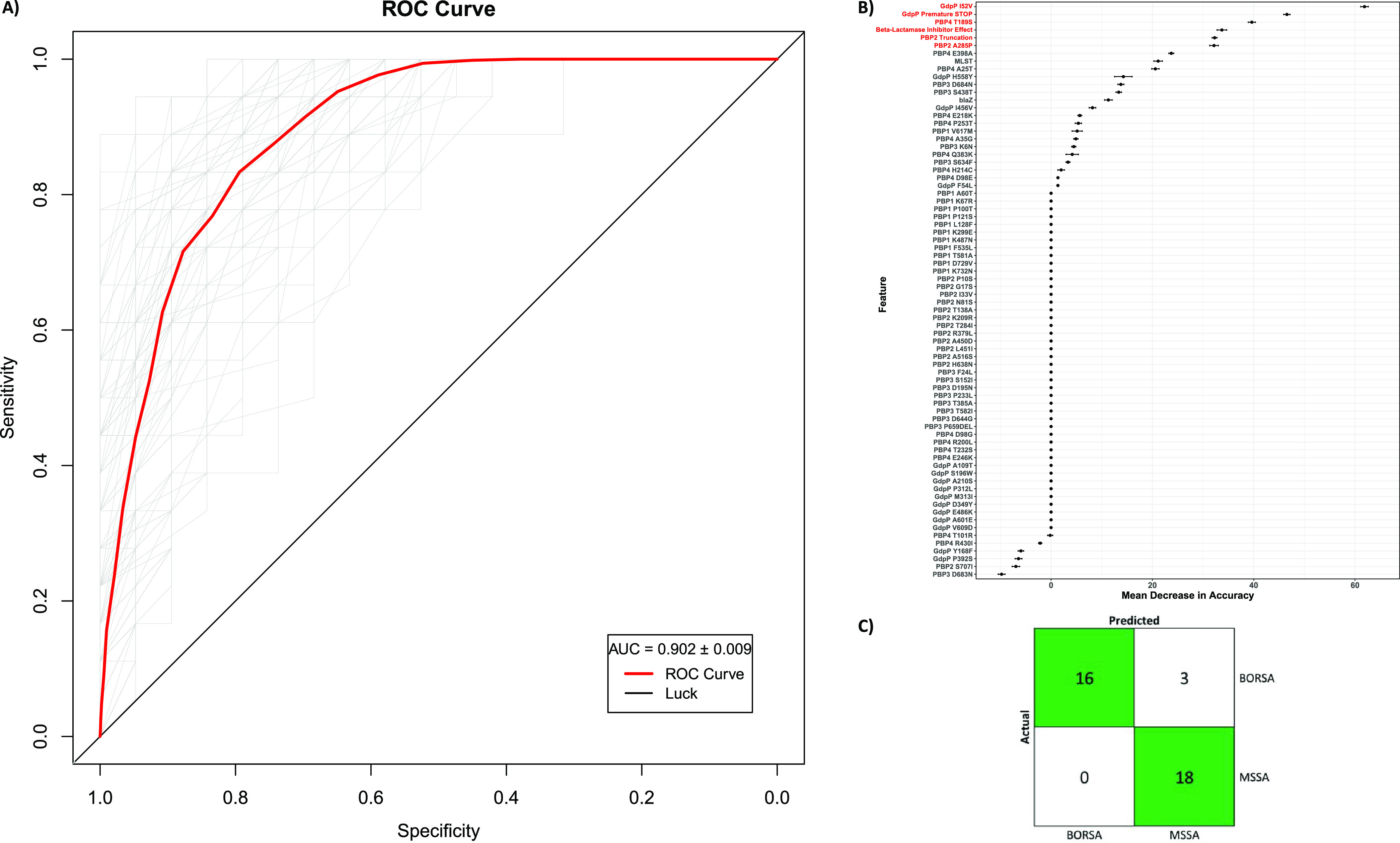
A random forest classifier predicts the BORSA phenotype by PBP/GdpP mutational profile with high diagnostic accuracy. (A) Receiver operating characteristic (ROC) curves evaluating the ability to predict the BORSA phenotype using random forest classification (RFC). The RFC was run over 100 iterations (denoted in gray) of the validation data set, each time randomly selected in a 70:30 train/test split, with the mean ROC curve in red. Each ROC curve represents the true-positive and false-positive rates of the supervised machine-learning model. (B) All remaining features used to train the prototype RFC after removal of highly correlated features, ranked by importance. The six features used in the final, sparse model (Fig. 5A) are signified in red. Data are means ± standard errors of the means computed over 100 iterations. (C) Confusion matrix for the classification of BORSA status using only these 6 predictors. The sparse model was highly accurate (91.9% classification accuracy).

We endeavored to parse the overlapping amino acid substitutional data to identify a detectable signature specific to our BORSA isolates. Isolates 307, 338, 340, and 342 were removed for this analysis, as sequencing data indicate that they are clones (≤10 whole-genome SNPs) of the first isolate taken from the same patient (*n* = 4) largely at the same sampling event (Fig. S4; Table S1). To increase our sample size, we incorporated an additional data set of 32 clinical BORSA isolates with published gene sequences for *pbp1* to *-4* and *gdpP*, as well as MLST and beta-lactamase inhibitor effect metadata (13) (Fig. S5A). Together with our curated BORSA and MSSA cohorts, this sample set was used to construct a supervised machine-learning model trained on the presence or absence of 123 unique amino acid substitutions across PBP1 to -4 and GdpP, truncations to PBP2 and/or GdpP, the presence of *blaZ*, MLST, and beta-lactamase inhibitor effect (Fig. S5B and C). We refined our random forest classifier (RFC) through a 2-fold feature elimination process in which 56 redundant features were first removed by correlation analysis. Of the remaining 72 features, 10-fold cross-validation indicated that just six are sufficient for an accurate prediction of BORSA status. We then trained a sparse RFC on the six most informative predictors (Fig. 5B) and achieved an average validation area under the receiver-operator curve (AUROC) of 0.902 ± 0.009 over 100 iterations (Fig. 5A). This model correctly classified 16 of 16 BORSA isolates and 18 of the 21 MSSA isolates (91.9% accuracy) (Fig. 5C). The top BORSA-correlated and anticorrelated features among S. aureus isolates within these cohorts were truncations to GdpP or PBP2, beta-lactamase inhibitor effect, and the amino acid substitutions GdpP I52V, PBP2 A285P, and PBP4 T189S (Fig. 5B; Table S4), representing a combination of lineage markers that allow accurate discrimination of BORSA and MSSA. Implementation of a phylogenetically informed association tool provided further support for the strong positive association of GdpP truncation with BORSA status, finding trait convergence over four independent genotype transitions within our cohort (Fig. S6). Our supervised machine-learning model trained on a subset of our cohort accurately identified whether an S. aureus isolate in the withheld data set was borderline oxacillin resistant or methicillin susceptible based on beta-lactamase hyperproduction, a truncated GdpP or PBP2, and three substitutions in PBP2 and PBP4, despite high-level heterogeneity in amino acid substitutions within canonical BORSA-associated proteins.

10.1128/mbio.03196-21.4 FIG S4 SNP distance of closely related isolates. (A to E) Outgroup-rooted whole-genome SNP distance trees of clonal isolates collected from the same inpatient on the same sampling date (A, C to E) and from the same inpatient over multiple sampling events (B). Dark-blue and sky-blue branch colors represent BORSA and MSSA statuses, respectively. All isolate pairs are ≤10 whole-genome SNPs in distance. (B) Isolates 332 and 338 were collected 7 days apart. Download FIG S4, PDF file, 0.09 MB. Copyright © 2022 Sawhney et al. 2022 Sawhney et al. https://creativecommons.org/licenses/by/4.0/ This content is distributed under the terms of the Creative Commons Attribution 4.0 International license.

10.1128/mbio.03196-21.5 FIG S5 Unrefined RFC built with inclusion of the Argudín et al. (13) data set. (A) BORSA mutations by protein from isolates in this and the Argudín et al. cohorts. (B, C) Prototype RFC preceding removal of highly correlated features. Data originally included all amino acid substitutions or truncations in the PBP1 to -4 and GdpP proteins, MLST, *blaZ* presence, and beta-lactamase inhibitor effect. Download FIG S5, PDF file, 0.5 MB. Copyright © 2022 Sawhney et al. 2022 Sawhney et al. https://creativecommons.org/licenses/by/4.0/ This content is distributed under the terms of the Creative Commons Attribution 4.0 International license.

10.1128/mbio.03196-21.6 FIG S6 Phylogenetically informed gene-wide mutation association analysis. (A) Manhattan plot of all genetic features (substitutions or truncations in PBP1 to -4 and GdpP) in 92 BORSA and MSSA isolates from this study, plotted against –ln(*P* value). Features in which the genotype is present or absent in all isolates or all but one isolate and features that do not display 2+ high-confidence genotype transition edges are excluded. (B) Genotype transitions of a GdpP premature stop codon are in red. (C) Null distribution and observed value of the significant hit “GdpP premature stop.” All plots were generated by hogwash. The significance threshold was set at an FDR-corrected *P* value of <0.05. Download FIG S6, PDF file, 0.08 MB. Copyright © 2022 Sawhney et al. 2022 Sawhney et al. https://creativecommons.org/licenses/by/4.0/ This content is distributed under the terms of the Creative Commons Attribution 4.0 International license.

10.1128/mbio.03196-21.10 TABLE S4 Bivariate association of the top random forest classifier features with BORSA and MSSA status. Significance was determined via Fisher’s exact test with FDR correction. Feature correlation and anticorrelation with the BORSA phenotype are noted. Download Table S4, DOCX file, 0.01 MB. Copyright © 2022 Sawhney et al. 2022 Sawhney et al. https://creativecommons.org/licenses/by/4.0/ This content is distributed under the terms of the Creative Commons Attribution 4.0 International license.

## DISCUSSION

Here, we report a pseudo-outbreak of suspected MRSA and an ensuing diagnostic and genomic characterization of 102 clinical S. aureus isolates of various oxacillin susceptibilities. These results confirm prior reports of diverse mutations in PBP proteins of BORSA isolates (11–13, 16, 17, 47, 51); however, the critical inclusion of a MSSA isolate bank from the same institution as comparators reveal that many of the previously reported BORSA-linked amino acid mutations from geographically distant collection sites are either not found in any of our cohorts or shared by BORSA and MSSA isolates alike. Given that we did not observe a core or accessory genome signature among BORSA isolates that was distinct from those of MSSA or MRSA isolates, we leveraged our sequencing data to construct a supervised machine-learning model with input features largely based on the mutational profile of historically BORSA-associated proteins. Through this work, we generated a random forest classifier trained on just six features that yielded a robust diagnostic accuracy for the detection of BORSA among MSSA isolates within our cohorts. Among these features are mutations and/or truncations to PBP2 and PBP4, a beta-lactamase hyperproducer effect, which was observed in some but not all our BORSA isolates, and a mutation in and truncation of the GdpP protein.

GdpP is a phosphodiesterase that catalyzes the hydrolysis of intracellular secondary messenger c-di-AMP, and S. aureus *gdpP* deletion mutants have been shown to have elevated c-di-AMP and PBP4 levels, thick cell walls, reduced cell size, and reduced susceptibility to beta-lactams and other cell wall-targeting antimicrobials (19, 20, 50, 52). Our study emphasizes its importance to the BORSA phenotype, as the sole feature exclusively present in certain isolates in our BORSA cohort and prior BORSA reports is a truncated GdpP protein [Fig. 4B, BORSA (Previously Reported) and BORSA (This Study) overlap]. Of significance, the lone BORSA clone among the cluster 2 isolates (Fig. 3B) harbors a premature stop codon in GdpP that is not present in any of the three MSSA clones that all together differ by a maximum of 17 whole-genome SNPs. Moreover, the two isolates collected from patient 33 are only 10 whole-genome SNPs apart (Fig. S4E), yet an SNP resulting in a premature stop codon is present in isolate 344 (BORSA) and absent in isolate 343 (MSSA). One hundred percent of the BORSA isolates that encode premature stop codons in their *gdpP* genes (*n* = 4) (Fig. 4A) were shown to be oxacillin resistant by both disk diffusion (zone size ≤ 10 mm) and gradient diffusion (Table 1). In addition, one of the three isolates with expected full-length GdpP proteins that also met this conservative criterion is the sole isolate in our cohort to harbor a D349Y substitution within the desert hedgehog (DHH) motif of GdpP, the domain responsible for c-di-AMP phosphodiesterase activity (19, 50). Taken together, our data demonstrate that a majority of isolates with the strongest BORSA phenotype (criteria i and ii) have truncated or mutated GdpP proteins, and one of the few discriminatory SNPs between nearly identical BORSA and MSSA isolates results in a premature stop codon in GdpP.

Many BORSA investigations utilize lab-adapted strains (11, 47, 50) or are multicenter studies (13, 16, 20). Among the few large-scale single-center studies (4, 53, 54), ours is remarkable as the first to indicate against a clonal outbreak within our institution, as supported by WGS of isolates. Indeed, we find BORSA isolates within our cohort to be spread across lineages and clades, phylogenetically dispersed among MSSA and MRSA isolates. Our data further support independent acquisition of borderline oxacillin resistance in a majority of our BORSA isolates, with infrequent vertical transfer of resistance from an immediate common ancestor. WGS was critical to these findings, yet there is a critical paucity of human-colonizing or -infecting BORSA isolate assemblies uploaded to the public NCBI GenBank database. Our work provides the addition of 33 high-quality BORSA assemblies for public use.

A challenge facing clinical microbiology laboratories is identifying and reporting BORSA due to the lack of a consensus definition and an established mechanism. This also complicates comparisons between studies and across previously published work. This work used several methods and two distinct BORSA definitions to help address these variabilities. A limitation of this study is that data were generated from a sole clinical microbiology laboratory. However, having a single laboratory perform these studies enabled us to standardize methods and better compare BORSA isolates to other S. aureus isolates in our microbiology region. In addition, isolates were collected from multiple hospitals and patient populations within our geographic area. Comparing our clinical investigation to previous studies, our findings are generalizable (11–13, 16, 17, 19, 47, 50, 51). Other limitations include the targeted isolate selection criteria and lack of BORSA prevalence data prior to this investigation.

A common challenge and limitation of machine-learning models is their potential to learn lineage markers rather than causal variants. Though we did not find MLST to be a strong contributor in the unrefined RFC (Fig. S5B) or find BORSA/MSSA statuses to be significantly different by lineage or sequence type, the potential influence of phylogenetic relatedness cannot be discounted. Given the lack of publicly available sequencing data available for BORSA isolates, we are unable to evaluate the robustness of our model for BORSA isolates beyond those included in this study. Further work reproducing our findings is therefore necessary before this sparse model can be considered generalizable.

MRSA screening can have significant impacts on patient care and clinical workflows. For example, MRSA-colonized infants are placed on contact precautions and may undergo decolonization regimens, such as intranasal mupirocin, to reduce the risk of subsequent infection. Misidentification of MRSA can lead to unnecessary use of isolation precautions and overuse of antimicrobial agents. In addition, MRSA screening is used to establish accurate hospital-acquired infection rates. In 2017, MRSA rates were included in the reimbursement equation in Medicare’s Hospital Value-Based Purchasing (VBP) program. This can have immense financial implications for hospitals, with penalties of a 1% reduction in payments if they are in the bottom 25%. With such high stakes on MRSA rates, it is critical to properly evaluate performance differences between MRSA screening methods. Nearly all molecular MRSA screening approaches look for the genomic markers *mecA* and/or *mecC*, yet BORSA is not *mecA* or *mecC* mediated. Though it is clear that BORSA detection varies by MRSA screening method and thus impacts MRSA rates, the degree of significance is dependent on BORSA prevalence, which is unknown at most institutions. Based on College of American Pathologists MRSA surveys in 2021, laboratories commonly use the following MRSA screening agars assessed in the presented work, all of which were assessed for MRSA and BORSA detection specificity and sensitivity here: BBL CHROMagar MRSA II (109 labs), chromID MRSA (56 labs), MRSA*Select* II (100 labs), HardyCHROM MRSA (8 labs), and Spectra MRSA (144 labs) agars.

The resistance mechanisms of the BORSA phenotype are complex and not fully understood. In this study, the phenotypic variability of BORSA isolates was highlighted by growth differences across screening agars, including the initial clinical MRSA surveillance testing. Within our cohort, one isolate (isolate 310) retested inconsistently (oxacillin gradient diffusion MIC = 2, 4, and 4 μg/mL on repeat testing). While it is unlikely that oxacillin would be used clinically for the treatment of infection with such an isolate, we chose to conservatively include isolate 310’s genome among the MSSA cohort due to its inconsistent borderline susceptibility to oxacillin and to be consistent with our in-text classification of BORSA as an organism for which the MIC is consistently ≥4 μg/mL. One explanation for variable “breakthroughs” is expression differences of resistance mechanisms within subpopulations. This breakthrough should not discourage the use of MRSA screening agars but should warrant follow-up testing.

The present work led to modification of clinical susceptibility testing of S. aureus isolates in our microbiology laboratory. Our laboratory uses customizable disk diffusion panels which now include both cefoxitin and oxacillin. Cefoxitin-susceptible isolates are evaluated for an oxacillin-nonsusceptible phenotype using CLSI 2007 breakpoints (susceptible is >12 mm; intermediate is 11 to 12 mm; resistant is <11 mm). Isolates consistent with BORSA are reported as such to aid in selection of antimicrobial therapy. This will also help our institution to determine BORSA prevalence and evaluate treatment outcomes for future studies. Screening agars may serve as a reasonable alternative, and clinical microbiology laboratories will likely continue to use screening agars to survey for resistant strains of S. aureus.

In summary, our work was prompted by the investigation of a possible MRSA outbreak in our NICU, which was determined to be nonclonal BORSA. We characterized this collection of isolates and subsequently established BORSA definitions based on phenotypic and genotypic findings, reevaluated commonly used MRSA screening agars, and implemented an accurate computational classifier which distinguishes BORSA from MSSA/MRSA using only six genetic and phenotypic features.

## MATERIALS AND METHODS

### Study cohort.

Institutional review board (IRB) approval was obtained for this study. MRSA surveillance cultures from anterior nares specimens of all NICU patients are performed as part of standard of care at St. Louis Children’s Hospital (SLCH) upon admission and weekly thereafter. Isolates from positive cultures are frozen as part of routine clinical protocols. Due to increased MRSA colonization rates in 2019, the SLCH Infection Prevention team reviewed all NICU patients with positive MRSA surveillance cultures and identified several cases with irregular results. The 32 cultures in question were taken from 31 subjects (one patient had two cultures that were evaluated); the median age of the subjects was 41 days, with an age range of 0 to 523 days. The microbiology laboratory at Barnes-Jewish Hospital reevaluated the NICU MRSA isolates using frozen stocks. Four of the 32 freezer stocks grew 2 colony morphologies, resulting in 36 isolates independently characterized from the NICU MRSA screening cultures. This investigation led to characterization of an additional 6 isolates that were collected from routine MRSA screening of non-NICU patients, which grew on MRSA screening agar despite being PBP2a negative. In total, there were 42 isolates under investigation (Table 1, isolates 301 to 346). This nonconsecutive order reflects the initial observation of different colony morphologies from freezer stocks that were later determined to be identical (see Fig. S4 in the supplemental material).

The 42 isolates under investigation were selected by subject matter experts in infection prevention who were concerned about unexpected positive culture results (i.e., the initial cohort represented an enriched sample set biased for an unusual phenotype). To put this enriched sample set into context, a second isolate collection was created using 50 consecutive cefoxitin-susceptible, PBP2a-negative S. aureus isolates (designated 1 to 50) recovered from blood cultures in the microbiology laboratory at Barnes-Jewish Hospital (this laboratory also serves SLCH) (Table 2). To further supplement this comparator collection, all S. aureus isolates recovered from blood cultures from patients in the NICU in 2019 (*n* = 10) were included (designated isolates 53 to 62). Taken together, the comparator blood isolates totaled 60, bringing the total sample set to 102 isolates.

### Identification and resistance characterization.

Microbial identification was confirmed by Bruker Biotyper MALDI-TOF MS (Bruker, Billerica, MA). For MRSA designation, a PBP2a SA culture colony test (Alere) was performed according to the manufacturer’s instructions using 18- to 24-h subculture growth. The presence of *mecA* and the homolog *mecC* were determined by in-house PCRs (22, 23). Susceptibility testing included that for cefoxitin by disk diffusion and for oxacillin by disk diffusion and gradient diffusion. Methods followed the procedural guidelines outlined by the Clinical and Laboratory Standards Institute (documents M02 and M23) (55, 56). Disk diffusion testing of cefoxitin (Hardy Diagnostics) and oxacillin (BD) was performed on conventional Mueller-Hinton agar (MHA) (Hardy Diagnostics). Gradient diffusion testing of oxacillin (bioMérieux) was performed on MHA with 2% NaCl agar (Remel). Detection of beta-lactamase production was assessed by the disk diffusion penicillin zone edge test (Hardy Diagnostics) and nitrocefin-based Cefinase disk test (Hardy Diagnostics). Beta-lactamase inhibitor rescue phenotype was determined by assessing the fold change in MIC from amoxicillin (bioMérieux) and amoxicillin-clavulanic acid (bioMérieux) gradient diffusion testing.

### MRSA screening agars.

Spectra MRSA screening agar (Remel) is routinely used by the Barnes-Jewish Hospital microbiology laboratory for MRSA active surveillance cultures. For clinical active surveillance cultures, anterior nares samples were collected using the BD E-Swab specimen collection and transport kit, plated to Spectra MRSA agar, and incubated/analyzed using the Kiestra laboratory automation system (BD) (57). Images were captured using the Kiestra system and modified by cropping and rotating for alignment. Five other commercially available MRSA screening agars were also used in this study: the chromID MRSA (bioMérieux), MRSA*Select* II (Bio-Rad), HardyCHROM MRSA (Hardy Diagnostics), BBL CHROMagar MRSA II (BD), and nonchromogenic MRSA screen plate (Hardy Diagnostics) agars. To compare growth differences, 18- to 24-h growth off blood agar plates was suspended at a 0.5 McFarland standard and 50 μL spotted to each agar. Two isolates were cultured per plate and struck for isolation, except with the nonchromogenic MRSA screen plate. For this, 8 isolates were cultured per plate by spotting 10 μL. The growth of all screening agars was analyzed after 18 to 24 h at 35°C in an air incubator. Images were modified by cropping, rotating for alignment, and adjusting modestly for contrast and brightness (images contain MRSA and MSSA isolates on the same plate/image for uniformity). Black lines were superimposed on some images to assist readers with differentiating strains on top and bottom.

### Illumina WGS.

Isolate DNA was extracted manually as previously described (58). DNA was quantified with the Quant-iT PicoGreen double-stranded DNA (dsDNA) assay (Thermo Fisher Scientific, Waltham, MA, USA). Isolate DNA at 0.5 ng was used as the input for Illumina library preparation using a Nextera kit (Illumina, San Diego, CA, USA). Libraries were pooled at equal concentrations and sequenced on the Illumina NextSeq 500 high-output platform (Illumina, San Diego, CA, USA) to a depth of 2.5 million reads per sample (2× 150 bp). Illumina adapter sequences were removed from demultiplexed reads using Trimmomatic (version 0.38) with the following parameters: leading, 10; trailing, 10; sliding window, 4:15; and minimum length (minlen), 60 (59). Potential human read contamination was removed using DeconSeq (version 0.4.3) with default parameters (60). Processed reads were then assembled into draft genomes using the *de novo* assembler SPAdes (version 3.13.0) with parameters –k 21,33,55,77 –careful (61). The scaffolds.fasta outputs were used for all downstream analyses. Draft genomes were determined to be >99% complete and <1% contaminated by CheckM (version 1.0.13), and assembly quality was calculated using QUAST (version 3.2) (62, 63). High-quality assemblies were annotated using Prokka (version 1.14) with a minimum contig length (–mincontiglen) of 500 to identify open reading frames (29).

### Core genome analysis.

The general feature format (.gff) files outputted by Prokka were used to construct a core genome alignment for all BORSA, MRSA, and MSSA isolates through Roary (version 3.12.0), with default parameters (30). The alignment, composed of 1,859 genes shared by all isolates at a minimum 95% identity, was used to generate an unrooted maximum likelihood tree with FastTree (version 2.1.10) (31). The resulting newick file was visualized in iTOL (32). *In silico* multilocus sequence typing (MLST) and *spa* typing were performed using the webtools MLST 2.0 and spaTyper 1.0, maintained by the Center for Genomic Epidemiology (64, 65). MLST, *spa* type, and class were viewed as color strips in iTOL. Lineages identified by hierBAPS during fastGEAR analysis were also marked on the trees (33). The ancestral state of borderline oxacillin resistance was estimated as a discrete trait using the maximum likelihood-based *ace* function (R ape package) (66).

### SNP distance determination.

The snp-sites (version 2.4.0) tool was used to call isolate-specific SNPs against the core genome alignment file created by Roary (67). A core genome SNP threshold of 600 was determined to be discriminatory for closely related isolates through an all-versus-all comparison, and isolate pairs with distances below this threshold were binned into clusters. Snippy (version 4.4.3) was then employed to call whole-genome SNPs between isolate pairs for each pair in the same cluster (68). Isolates were considered clones of the same persisting strain if their whole-genome SNP distance (Snippy VariantTotal) was below 30 (≥99.999% average nucleotide identity [ANI]) (69). Rooted SNP distance trees were created with the R ape package.

### Accessory genome analysis.

The gene_presence_absence.Rtab output file from Roary was purged of core genes and used to calculate the Jaccard distance between all isolates through the vegdist function (R vegan package) (70). Clustering by accessory gene content was visualized through principal-coordinate analysis using the pcoa and ggplot functions (R ape and ggplot2 packages) (66, 71).

Antibiotic resistance genes (ARGs) were initially annotated *in silico* against the NCBI comprehensive database of acquired and intrinsic antimicrobial resistance proteins at >90% identity using AMRFinder (version 3.8.4) (72). A presence-absence matrix was generated using the pheatmap function (R pheatmap and dendsort packages) (73, 74), in which isolates were clustered by ARG presence, with associated metadata displayed as color strips to represent isolate class, sequence type, and expected antimicrobial resistance (aminoglycoside, beta-lactams, fosfomycin, fusidic acid, lincosamide, macrolide, mupirocin, quaternary ammonium, and tetracycline).

### Selected protein investigation.

The PBP1 to -4 and GdpP proteins were chosen for further investigation based on published literature (12, 13, 16, 17, 19). Amino acid sequences with the following headers were extracted from Prokka .faa output files: penicillin-binding protein, d-alanyl-d-alanine carboxypeptidase, and cyclic-di-AMP phosphodiesterase GdpP. The PBP1 to -4 and GdpP amino acid sequences from 32 published BORSA isolates (13) were also incorporated (GenBank accession no. MF070915.1 to MF071042.2 and MF071075.1 to MF071106.1). Sequences alignments for each protein were performed using Clustal Omega and visualized with Jalview (48, 49). All substitutions, deletions, and protein truncations against the consensus sequence were manually curated. Results were compared against those found in previous BORSA reports (11–13, 16, 17, 19, 47, 50, 51), with overlap visualized with the Euler function (R eulerr package) (75).

### Random forest classification of the BORSA phenotype.

A custom machine-learning process employing random forest analysis was used to distinguish between the aforementioned BORSA and MSSA isolates. The initial model consisted of 128 features, including amino acid mutations in the PBP1 to -4 and GdpP proteins, the presence of *blaZ*, MLST, and the beta-lactamase inhibitor effect, and was trained on 70% of the isolate data set, with 30% withheld for validation. The randomForest function (R randomForest package) was utilized at default parameters with the following adjustments to establish a baseline model for optimization: ntree=5,000, proximity=FALSE, importance=TRUE. A feature elimination step was then performed to improve the performance of the subsequent model iterations. To minimize redundancy, we used the cor and findCorrelation functions (R STATS, caret packages) to calculate Spearman correlation coefficients across all features, with highly correlated features determined as those with >0.75 correlation (76, 77). A representative of each set of correlated features was retained, along with all uncorrelated features. The rfeControl and rfe functions (R caret package) were used to perform 10-fold cross-validations on the 72 remaining features to estimate the minimum number of RFC features required to optimally predict the BORSA phenotype (77). The most important features for phenotype classification were identified from the mean decrease in accuracy index as determined over 100 iterations of the importance function (R randomForest package) on the decorrelated classifier (78). The top six features as determined by accuracy index were used to build a sparse model with the following parameters: ntree=5,000, mtry=3, proximity=FALSE, importance=TRUE. These features included 5 amino acid substitutions or protein truncations in PBP2, PBP4, and GdpP and beta-lactamase inhibitor effect. The sparse model was run over 100 iterations of the 70/30 training/validation data set split, and model performance was measured through the widely used AUROC (area under the receiver-operator curve) estimator using the prediction and performance functions (R ROCR package) at default parameters (79). The mean validation AUROC value was reported with 95% confidence intervals. The ROC plot was visualized using the predict and roc functions (R pROC package) at default parameters (80). A phylogenetically informed gene-wide mutation association analysis was performed with hogwash (default parameters, FDR = 0.05) on only BORSA and MSSA isolates from our cohort (*n* = 92) (81).

### Statistical analysis.

Comparisons of categorical and continuous data were performed by Fisher’s exact test with FDR correction and the Mann-Whitney U test, respectively. All tests were two tailed, and statistical significance was defined as a *P *of *≤*0.05 (*) and a *P *of *≤*0.01 (**).

### Data availability.

All isolate assemblies are available in NCBI GenBank under BioProject accession no. PRJNA695316. All RFC-relevant code is publicly available at https://github.com/sanjsawhney/BORSA_RFC.
