# Supplementary material for: Comparative Genomics of Borderline Oxacillin-Resistant Staphylococcus aureus Detected during a Pseudo-outbreak of Methicillin-Resistant S. aureus in a Neonatal Intensive Care Unit
Source: mBio. 2022 Jan 18;13(1):e03196-21. doi: 10.1128/mbio.03196-21 (PMC8764539; doi:10.1128/mbio.03196-21)
Supplement: TABLE S1 [file mbio.03196-21-st001.docx]

**Table S1: Compiled data on all isolates.** Isolates originally under investigation and comparator blood isolates are numbered 301-346 and 1-62, respectively.

| Isolate |  |  | PBP2a | *mecA* PCR | *mecC* PCR | Cefoxitin DD, MH | | Oxacillin DD, MH | | Oxacillin, GD  2% NaCl MH | | Pen DD Zone-edge Test | | Cefinase Test | Beta-lactamase Inhibitor Impact (fold) | MRSA Screening Agars | | | | | |
| --- | --- | --- | --- | --- | --- | --- | --- | --- | --- | --- | --- | --- | --- | --- | --- | --- | --- | --- | --- | --- | --- |
|  | Class | Patient |  |  |  | Zone  (mm) | I | Zone (mm) | I | MIC | I |  |  |  |  | Spectra  MRSA | | HardyCHROM MRSA | Nonchromogenic MRSA Screen Agar | | Other 3 Agars* |
| 301 | BORSA | 1 | - | - | - | 24 | S | 6 | R | 16 | R | P | P | | 2 | +++ | +++ | | +++ | - | |
| 302 | BORSA | 2 | - | - | - | 24 | S | 11 | I | 1 | S | P | P | | 4 | +++ | +++ | | - | - | |
| 303 | MRSA | 3 | P | Det | - | 11 | R | 6 | R | >256 | R | P | P | | 4 | +++ | +++ | | +++ | +++ | |
| 304 | MSSA | 4 | - | - | - | 25 | S | 21 | S | 1 | S | - | - | | 1 | + | - | | - | - | |
| 305 | BORSA | 4 | - | - | - | 24 | S | 10 | R | 4 | R | P | P | | 2 | ++ | +++ | | - | - | |
| 306 | BORSA | 5 | - | - | - | 24 | S | 18 | S | 8 | R | P | P | | 2 | ++ | +++ | | - | - | |
| 307 | BORSA | 5 | - | - | - | 25 | S | 15 | S | 4 | R | P | P | | 2 | ++ | - | | - | - | |
| 308 | MRSA | 6 | P | Det | - | 10 | R | 6 | R | >256 | R | P | - | | 1 | +++ | +++ | | +++ | +++ | |
| 309 | MRSA | 7 | P | Det | - | 11 | R | 6 | R | >256 | R | P | P | | 8 | +++ | +++ | | +++ | +++ | |
| 310 | BORSA/MSSA^#^ | 8 | - | - | - | 27 | S | 16 | S | 4 | R | P | P | | 1 | ++ | - | | - | - | |
| 311 | MSSA | 9 | - | - | - | 25 | S | 15 | S | 2 | S | P | P | | 1 | ++ | ++ | | - | - | |
| 312 | MSSA | 10 | - | - | - | 24 | S | 17 | S | 2 | S | P | P | | 1 | ++ | - | | - | - | |
| 314 | MSSA | 11 | - | - | - | 27 | S | 22 | S | 1 | S | - | - | | 1 | +++ | - | | - | - | |
| 315 | BORSA | 12 | - | - | - | 25 | S | 16 | S | 16 | R | P | P | | 2 | ++ | - | | - | - | |
| 316 | BORSA | 13 | - | - | - | 27 | S | 16 | S | 4 | R | P | P | | 4 | + | - | | - | - | |
| 318 | BORSA | 14 | - | - | - | 27 | S | 10 | R | 8 | R | P | P | | 2 | + | - | | - | - | |
| 320 | MRSA | 15 | P | Det | - | 10 | R | 6 | R | >256 | R | P | P | | 8 | +++ | +++ | | +++ | +++ | |
| 321 | BORSA | 16 | - | - | - | 23 | S | 6 | R | 16 | R | P | P | | 2 | +++ | +++ | | +++ | - | |
| 322 | MRSA | 16 | P | Det | - | 6 | R | 6 | R | >256 | R | P | P | | 16 | +++ | +++ | | +++ | +++ | |
| 323 | BORSA | 17 | - | - | - | 24 | S | 13 | S | 4 | R | P | P | | 4 | +++ | ++ | | +++ | - | |
| 324 | MRSA | 18 | P | Det | - | 14 | R | 10 | R | >256 | R | - | - | | 1 | +++ | +++ | | +++ | +++ | |
| 325 | MSSA | 19 | - | - | - | 25 | S | 17 | S | 2 | S | P | P | | 2 | + | - | | - | - | |
| 327 | BORSA | 20 | - | - | - | 28 | S | 15 | S | 8 | R | P | P | | 4 | +++ | - | | - | - | |
| 328 | BORSA | 21 | - | - | - | 24 | S | 11 | I | 4 | R | P | P | | 2 | ++ | - | | - | - | |
| 329 | MRSA | 22 | P | Det | - | 18 | R | 8 | R | 16 | R | P | P | | 2 | +++ | ++ | | +++ | ++ | |
| 330 | MRSA | 23 | P | Det | - | 12 | R | 6 | R | >256 | R | P | P | | 4 | +++ | + | | +++ | +++ | |
| 331 | MSSA | 24 | - | - | - | 24 | S | 16 | S | 2 | S | P | P | | 2 | ++ | - | | - | - | |
| 332 | BORSA | 25^ | - | - | - | 24 | S | 16 | S | 4 | R | P | P | | 2 | + | - | | - | - | |
| 333 | MSSA | 26 | - | - | - | 30 | S | 18 | S | 2 | S | P | P | | 2 | ++ | - | | - | - | |
| 334 | BORSA | 27 | - | - | - | 27 | S | 15 | S | 8 | R | P | P | | 2 | + | - | | - | - | |
| 335 | MRSA | 28 | P | Det | - | 25 | S | 12 | I | 2 | S | - | - | | 1 | ++ | - | | +++ | - | |
| 336 | BORSA | 29 | - | - | - | 25 | S | 10 | R | 16 | R | P | P | | 2 | + | ++ | | ++ | - | |
| 337 | BORSA | 30 | - | - | - | 26 | S | 14 | S | 4 | R | P | P | | 4 | +++ | + | | ++ | - | |
| 338 | BORSA | 25^ | - | - | - | 25 | S | 17 | S | 4 | R | P | P | | 2 | + | - | | ++ | - | |
| 339 | BORSA | 31 | - | - | - | 22 | S | 16 | S | 4 | R | P | P | | 4 | +++ | +++ | | ++ | - | |
| 340 | BORSA | 30 | - | - | - | 23 | S | 16 | S | 8 | R | P | P | | 2 | +++ | ++ | | ++ | - | |
| 341 | BORSA | 32 | - | - | - | 22 | S | 18 | S | 8 | R | P | P | | 2 | ++ | ++ | | ++ | - | |
| 342 | BORSA | 32 | - | - | - | 22 | S | 18 | S | 8 | R | P | P | | 2 | ++ | ++ | | ++ | - | |
| 343 | MSSA | 33 | - | - | - | 22 | S | 19 | S | 2 | S | P | P | | 4 | - | ++ | | - | - | |
| 344 | BORSA | 33 | - | - | - | 19 | R | 6 | R | 32 | R | P | P | | 2 | +++ | +++ | | ++ | - | |
| 345 | BORSA | 34 | - | - | - | 22 | S | 14 | S | 16 | R | P | P | | 2 | +++ | - | | - | - | |
| 346 | BORSA | 35 | - | - | - | 19 | R | 6 | R | 16 | R | P | P | | 2 | +++ | +++ | | ++ | - | |
| 1 | MSSA | 36 | - | - | - | 27 | S | 17 | S | 0.5 | S | P | P | | 4 | - | - | | - | - | |
| 2 | MSSA | 37 | - | - | - | 25 | S | 22 | S | 1 | S | - | - | | 1 | - | - | | - | - | |
| 3 | MSSA | 38 | - | - | - | 26 | S | 21 | S | 2 | S | - | - | | 1 | - | - | | - | - | |
| 4 | MSSA | 39 | - | - | - | 26 | S | 17 | S | 0.5 | S | P | P | | 2 | - | - | | - | - | |
| 5 | MSSA | 40 | - | - | - | 25 | S | 16 | S | 1 | S | P | P | | 2 | - | - | | - | - | |
| 6 | MSSA | 41 | - | - | - | 23 | S | 15 | S | 2 | S | P | P | | 2 | - | - | | - | - | |
| 7 | MSSA | 42 | - | - | - | 26 | S | 19 | S | 0.5 | S | P | P | | 2 | - | - | | - | - | |
| 8 | MSSA | 43 | - | - | - | 24 | S | 16 | S | 1 | S | P | P | | 2 | - | - | | - | - | |
| 9 | MSSA | 44 | - | - | - | 26 | S | 14 | S | 2 | S | P | P | | 2 | - | - | | - | - | |
| 10 | BORSA | 45 | - | - | - | 24 | S | 13 | S | 4 | R | P | P | | 2 | - | - | | - | - | |
| 11 | MSSA | 46 | - | - | - | 26 | S | 19 | S | 1 | S | P | P | | 2 | + | - | | - | - | |
| 12 | BORSA | 47 | - | - | - | 22 | S | 13 | S | 4 | R | P | P | | 2 | - | ++ | | - | - | |
| 13 | MSSA | 48 | - | - | - | 26 | S | 21 | S | 2 | S | P | P | | 1 | - | - | | - | - | |
| 14 | MSSA | 49 | - | - | - | 27 | S | 24 | S | 0.5 | S | - | - | | 1 | - | - | | - | - | |
| 15 | MSSA | 50 | - | - | - | 26 | S | 20 | S | 2 | S | - | - | | 1 | - | - | | - | - | |
| 16 | MSSA | 51 | - | - | - | 26 | S | 22 | S | 0.5 | S | - | - | | 1 | - | - | | - | - | |
| 17 | MSSA | 52 | - | - | - | 26 | S | 21 | S | 1 | S | - | - | | 1 | - | - | | - | - | |
| 18 | MSSA | 53 | - | - | - | 25 | S | 17 | S | 1 | S | P | P | | 2 | - | - | | - | - | |
| 19 | MSSA | 54 | - | - | - | 25 | S | 16 | S | 2 | S | P | P | | 2 | - | - | | - | - | |
| 20 | BORSA | 55 | - | - | - | 25 | S | 15 | S | 4 | R | P | P | | 2 | - | - | | - | - | |
| 21 | MSSA | 56 | - | - | - | 26 | S | 15 | S | 2 | S | P | P | | 2 | - | - | | - | - | |
| 22 | MSSA | 57 | - | - | - | 23 | S | 16 | S | 2 | S | P | P | | 2 | - | ++ | | - | - | |
| 23 | BORSA | 58 | - | - | - | 26 | S | 16 | S | 8 | R | P | P | | 2 | - | + | | - | - | |
| 24 | MSSA | 59 | - | - | - | 24 | S | 17 | S | 1 | S | P | P | | 2 | - | - | | - | - | |
| 25 | MSSA | 60 | - | - | - | 24 | S | 17 | S | 2 | S | P | P | | 2 | - | - | | - | - | |
| 26 | BORSA | 61 | - | - | - | 25 | S | 20 | S | 4 | R | - | - | | 1 | ++ | - | | + | - | |
| 27 | MSSA | 62 | - | - | - | 27 | S | 20 | S | 2 | S | - | - | | 1 | - | - | | - | - | |
| 28 | MSSA | 63 | - | - | - | 28 | S | 21 | S | 2 | S | - | - | | 1 | - | - | | - | - | |
| 29 | BORSA | 64 | - | - | - | 24 | S | 13 | S | 8 | R | P | P | | 2 | - | - | | - | - | |
| 30 | MSSA | 65 | - | - | - | 24 | S | 18 | S | 0.5 | S | P | P | | 2 | - | - | | - | - | |
| 31 | MSSA | 66 | - | - | - | 26 | S | 18 | S | 1 | S | P | P | | 2 | - | - | | - | - | |
| 32 | MSSA | 67 | - | - | - | 24 | S | 14 | S | 2 | S | P | P | | 2 | - | - | | - | - | |
| 33 | MSSA | 68 | - | - | - | 27 | S | 17 | S | 1 | S | P | P | | 4 | - | - | | - | - | |
| 34 | BORSA | 69 | - | - | - | 25 | S | 14 | S | 8 | R | P | P | | 2 | - | - | | - | - | |
| 35 | MSSA | 70 | - | - | - | 26 | S | 20 | S | 1 | S | - | - | | 1 | - | - | | - | - | |
| 36 | MSSA | 71 | - | - | - | 25 | S | 21 | S | 1 | S | - | - | | 1 | - | - | | - | - | |
| 37 | BORSA | 72 | - | - | - | 30 | S | 15 | S | 8 | R | P | P | | 2 | - | - | | - | - | |
| 38 | MSSA | 73 | - | - | - | 27 | S | 19 | S | 0.5 | S | P | P | | 2 | - | - | | - | - | |
| 39 | MSSA | 74 | - | - | - | 28 | S | 19 | S | 0.25 | S | P | P | | 2 | - | - | | - | - | |
| 40 | MSSA | 75 | - | - | - | 29 | S | 21 | S | 2 | S | - | - | | 1 | - | - | | - | - | |
| 41 | MSSA | 76 | - | - | - | 22 | S | 18 | S | 2 | S | - | - | | 1 | - | - | | - | - | |
| 42 | MSSA | 77 | - | - | - | 25 | S | 16 | S | 1 | S | P | P | | 2 | - | - | | - | - | |
| 43 | MSSA | 78 | - | - | - | 27 | S | 18 | S | 1 | S | P | P | | 2 | - | - | | - | - | |
| 44 | MSSA | 79 | - | - | - | 28 | S | 17 | S | 2 | S | P | P | | 2 | - | - | | - | - | |
| 45 | MSSA | 80 | - | - | - | 26 | S | 15 | S | 2 | S | P | P | | 2 | - | - | | - | - | |
| 46 | MSSA | 81 | - | - | - | 25 | S | 16 | S | 1 | S | P | P | | 2 | - | - | | - | - | |
| 47 | MSSA | 82 | - | - | - | 26 | S | 21 | S | 2 | S | - | - | | 1 | - | - | | - | - | |
| 48 | MSSA | 83 | - | - | - | 25 | S | 14 | S | 2 | S | P | P | | 2 | - | - | | - | - | |
| 49 | BORSA | 84 | - | - | - | 25 | S | 16 | S | 16 | R | P | P | | 2 | - | - | | - | - | |
| 50 | MSSA | 85 | - | - | - | 29 | S | 17 | S | 1 | S | P | P | | 2 | - | - | | - | - | |
| 53 | MSSA | 86 | - | - | - | 23 | S | 14 | S | 2 | S | P | P | | 4 | - | - | | - | - | |
| 54 | MSSA | 87 | - | - | - | 23 | S | 14 | S | 2 | S | P | P | | 2 | + | - | | - | - | |
| 55 | MSSA | 88 | - | - | - | 24 | S | 17 | S | 1 | S | P | P | | 2 | - | - | | - | - | |
| 56 | MSSA | 89 | - | - | - | 26 | S | 26 | S | 0.25 | S | - | P | | 1 | - | - | | - | - | |
| 57 | MSSA | 90 | - | - | - | 24 | S | 19 | S | 1 | S | P | P | | 4 | - | - | | - | - | |
| 58 | MSSA | 91 | - | - | - | 25 | S | 23 | S | 1 | S | - | - | | 1 | - | - | | - | - | |
| 59 | MSSA | 92 | - | - | - | 24 | S | 18 | S | 2 | S | P | P | | 2 | ++ | - | | - | - | |
| 60 | MSSA | 93 | - | - | - | 24 | S | 16 | S | 2 | S | P | P | | 4 | - | - | | - | - | |
| 61 | MSSA | 94 | - | - | - | 25 | S | 20 | S | 2 | S | P | P | | 1 | - | - | | - | - | |
| 62 | MSSA | 95 | - | - | - | 24 | S | 17 | S | 1 | S | P | P | | 2 | ++ | - | | - | - | |

Abbreviations: DD, disk diffusion; GD, gradient diffusion; MH, Mueller-Hinton agar; P, positive; -, negative, not detected, or no growth; Det, detected; S, susceptible; I, intermediate; R, resistant. Plus signs indicate growth abundance: rare (+), few (++), or growth equal to control strain (+++). ^#^Isolate shared characteristics consistent with BORSA and MSSA (see Discussion for details). ^Isolates from two separate cultures but same patient. *The agars MRSASelect II, BBL CHROMagar MRSA II, and chromID MRSA performed identically.
