# Supplementary figures and images for: Comparative Genomics of Borderline Oxacillin-Resistant Staphylococcus aureus Detected during a Pseudo-outbreak of Methicillin-Resistant S. aureus in a Neonatal Intensive Care Unit
Source: mBio. 2022 Jan 18;13(1):e03196-21. doi: 10.1128/mbio.03196-21 (PMC8764539; doi:10.1128/mbio.03196-21)

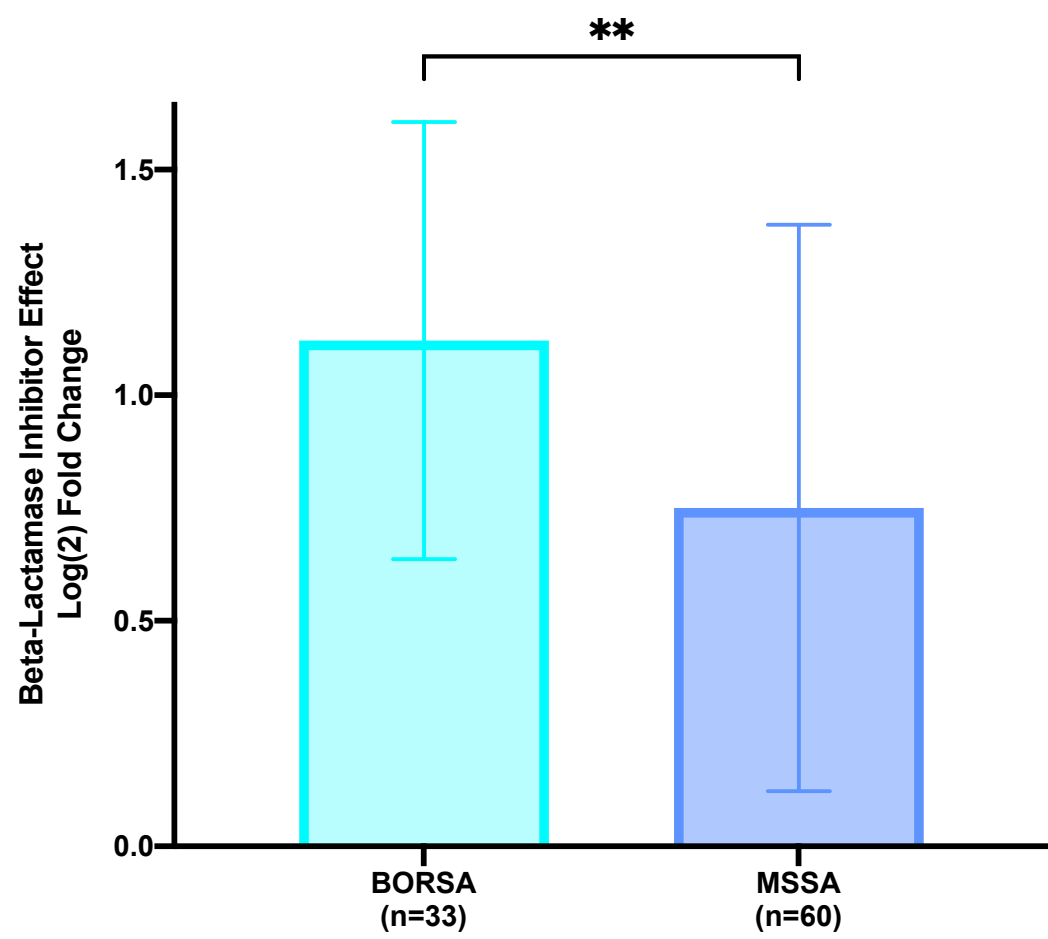

Supplement: FIG S1 [file mbio.03196-21-sf001.pdf]

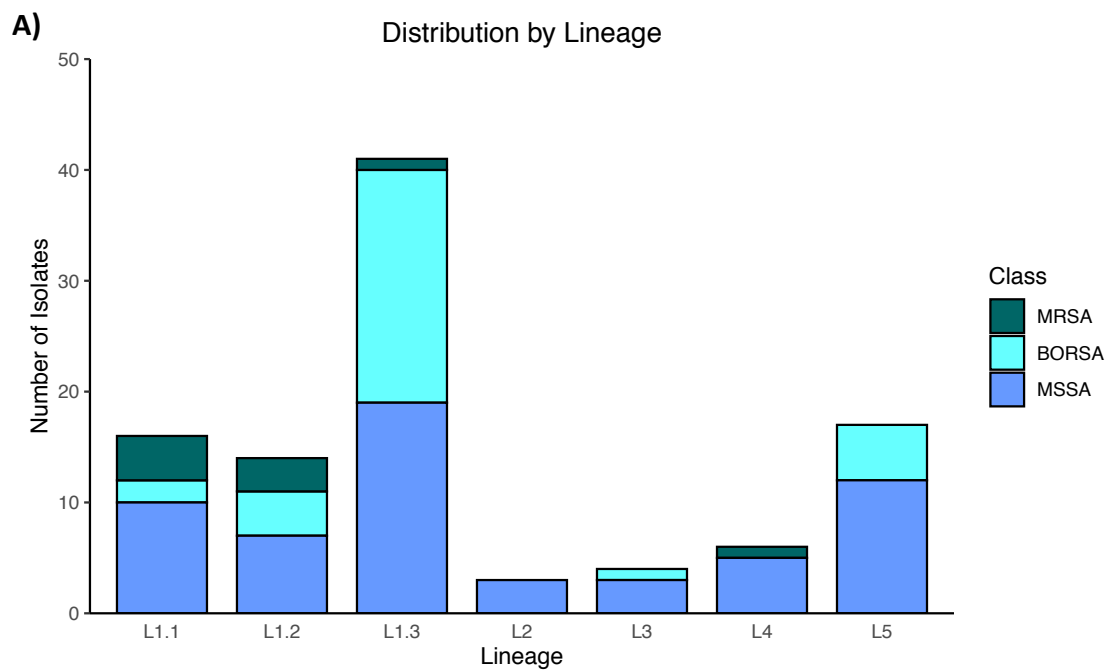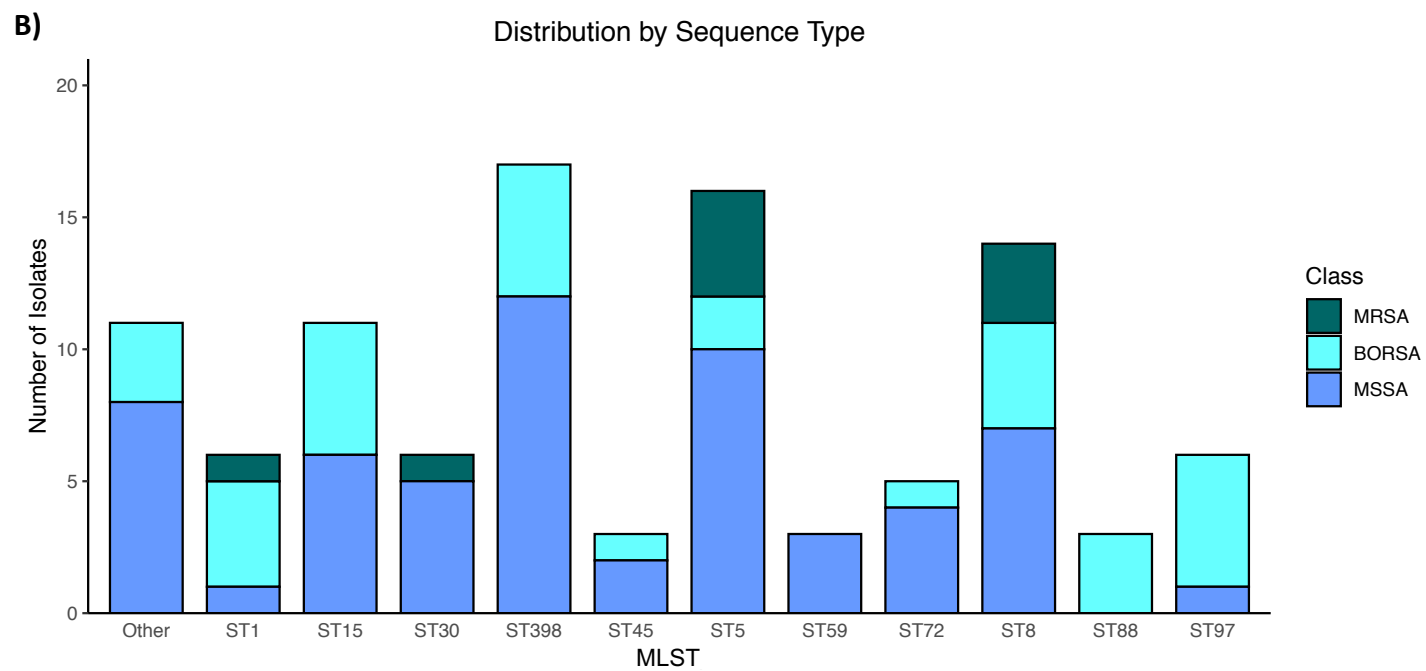

Supplement: FIG S2 [file mbio.03196-21-sf002.pdf]

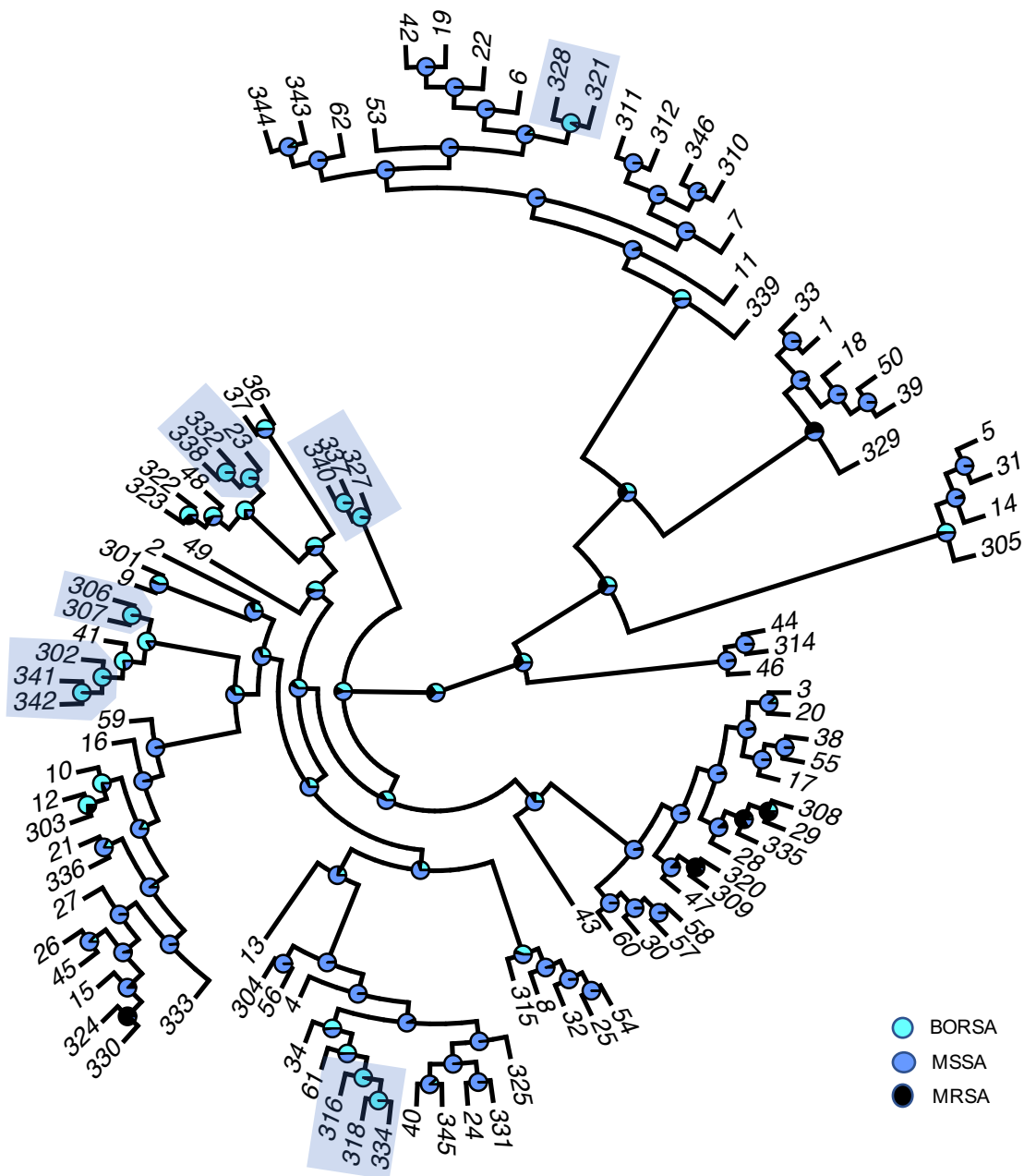

Supplement: FIG S3 [file mbio.03196-21-sf003.pdf]

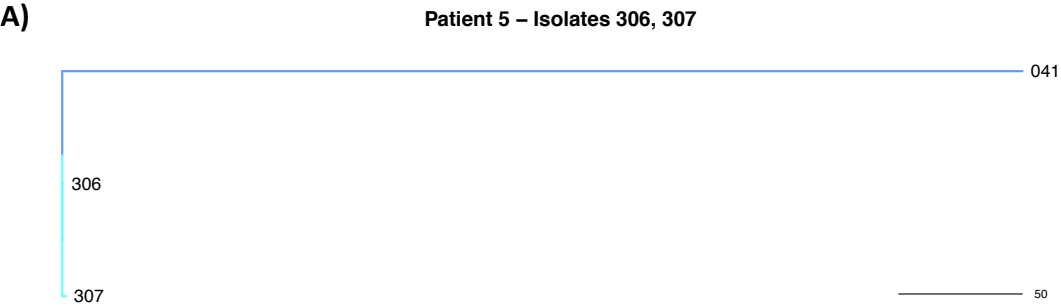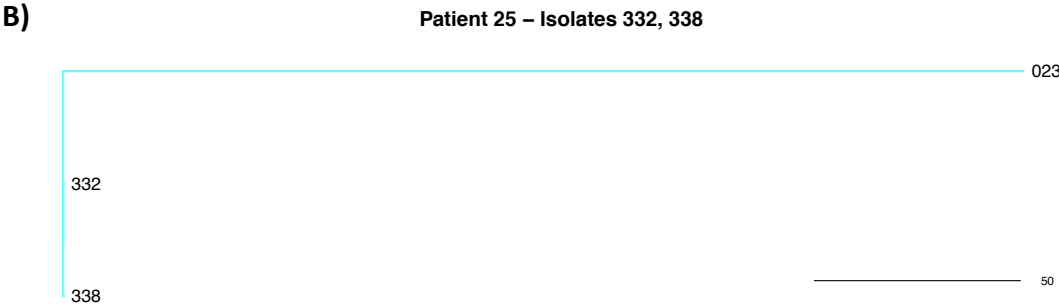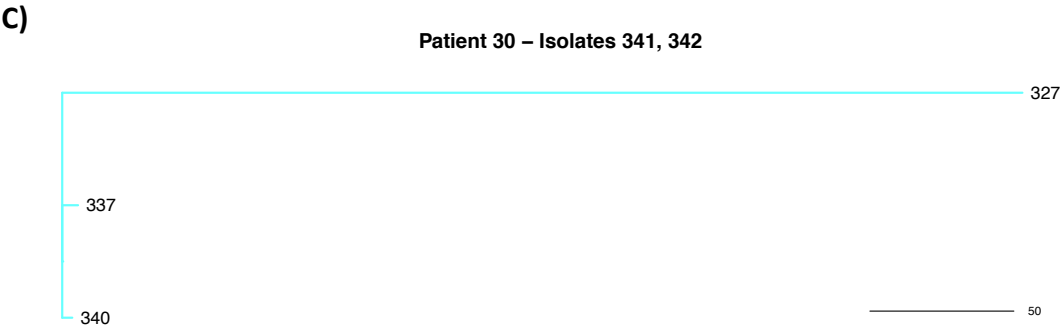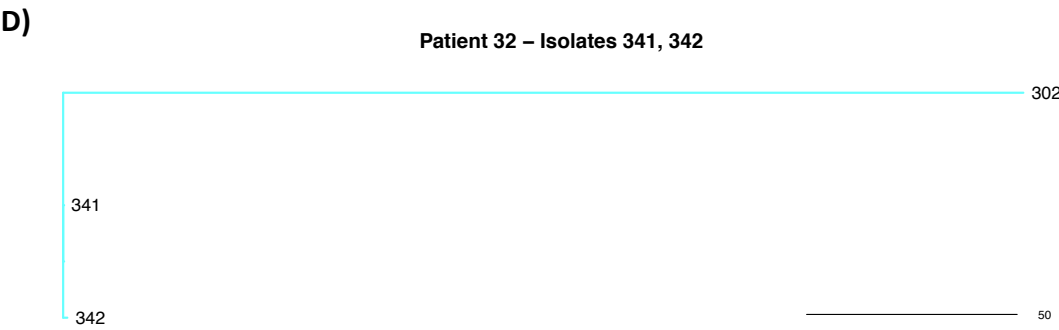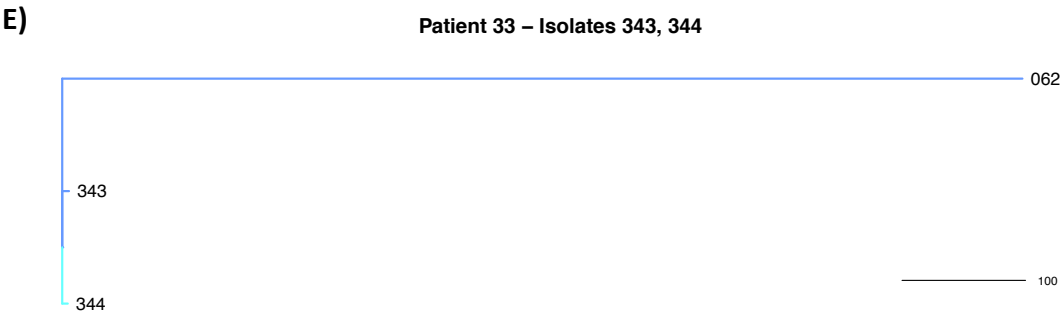

Supplement: FIG S4 [file mbio.03196-21-sf004.pdf]

**A)**

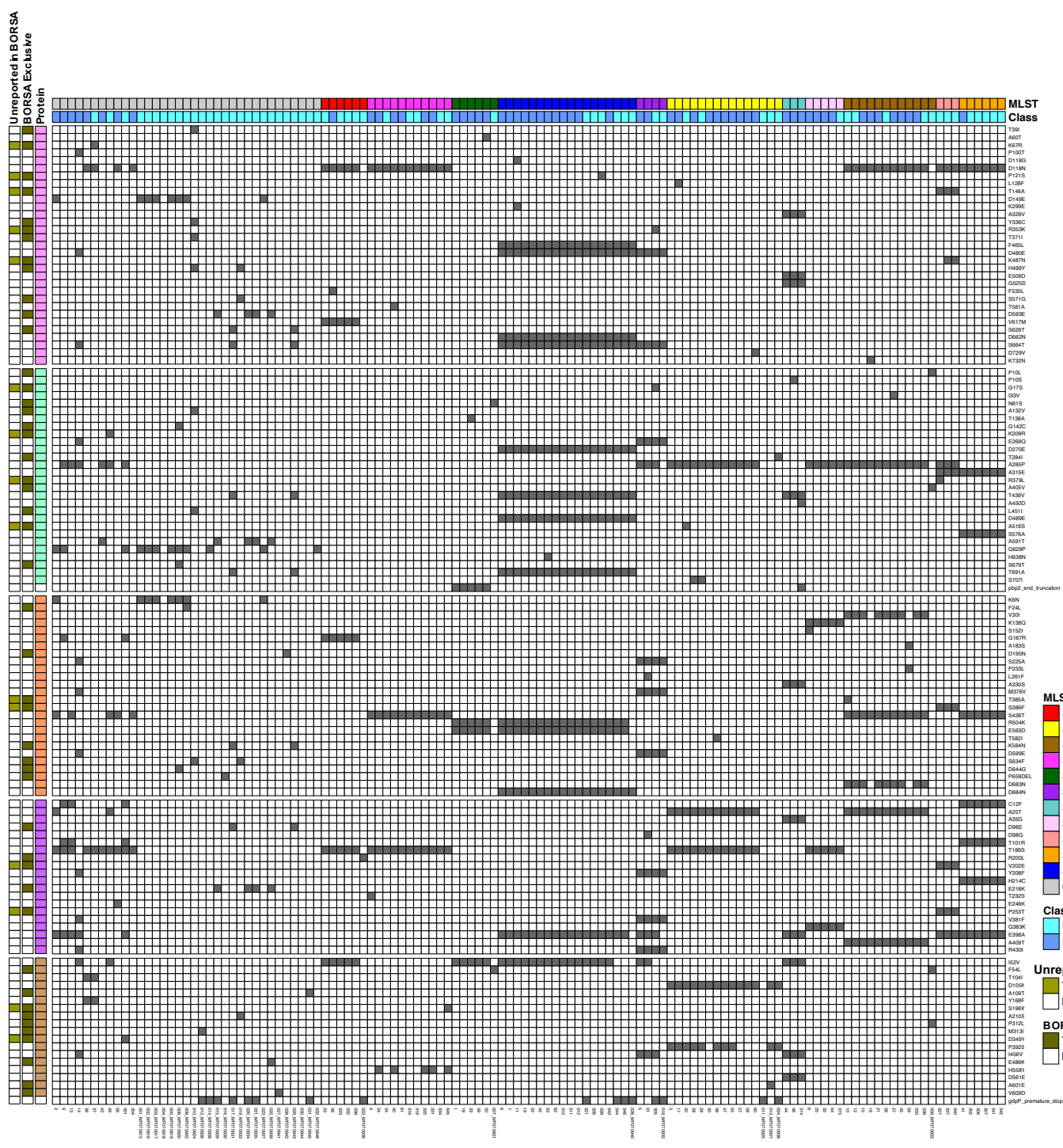

**B'**

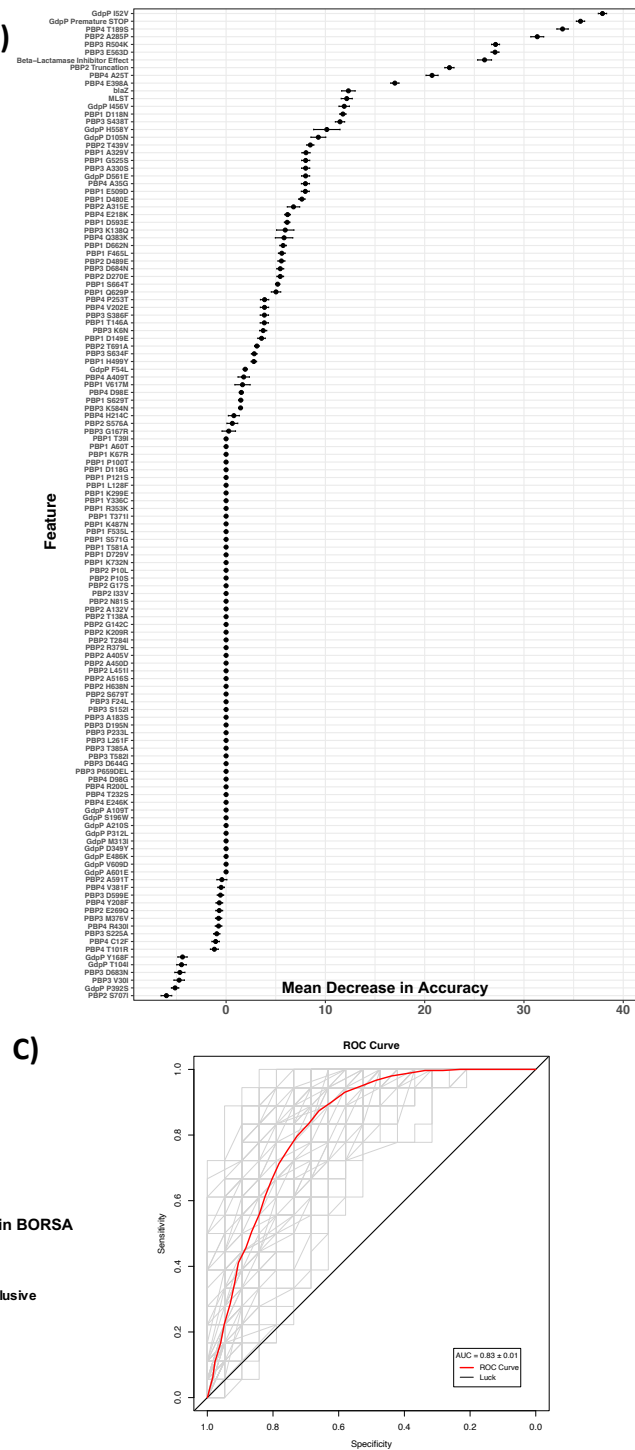

Supplement: FIG S5 [file mbio.03196-21-sf005.pdf]

A)

PhyC Test: hogwash

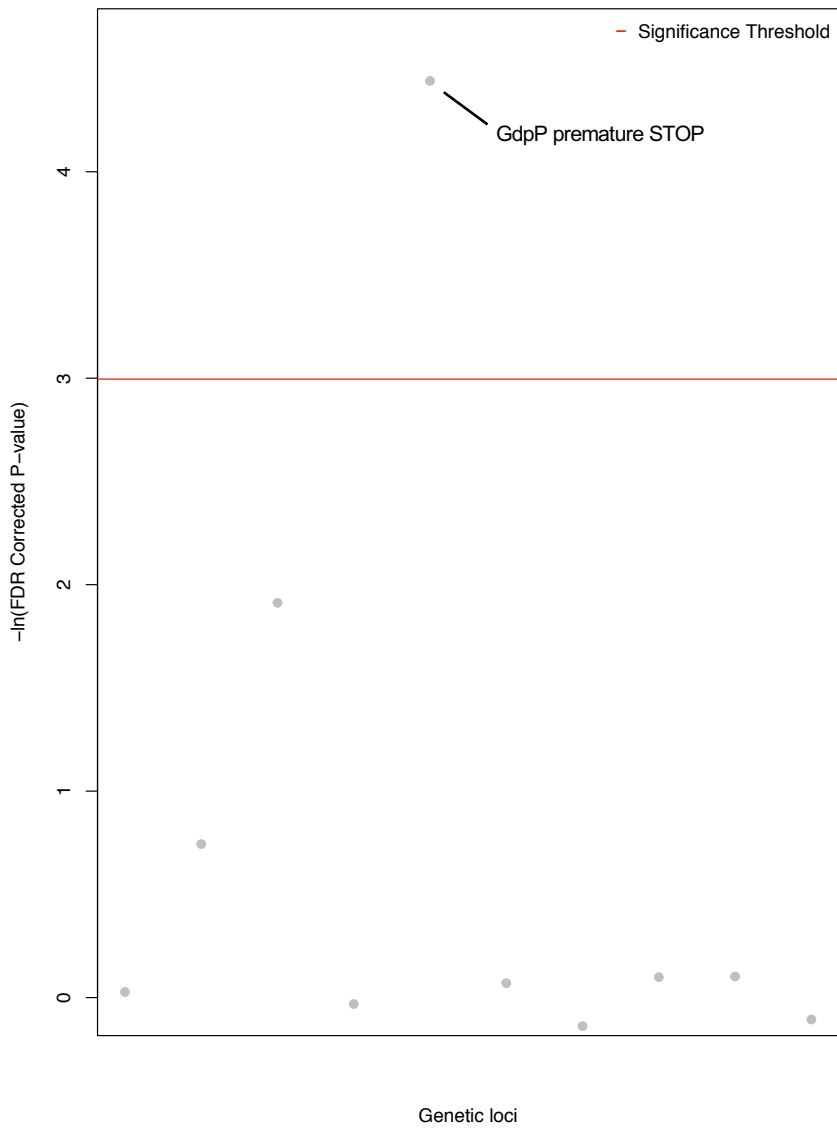

B)

GdpP premature STOP  
Genotype Transitions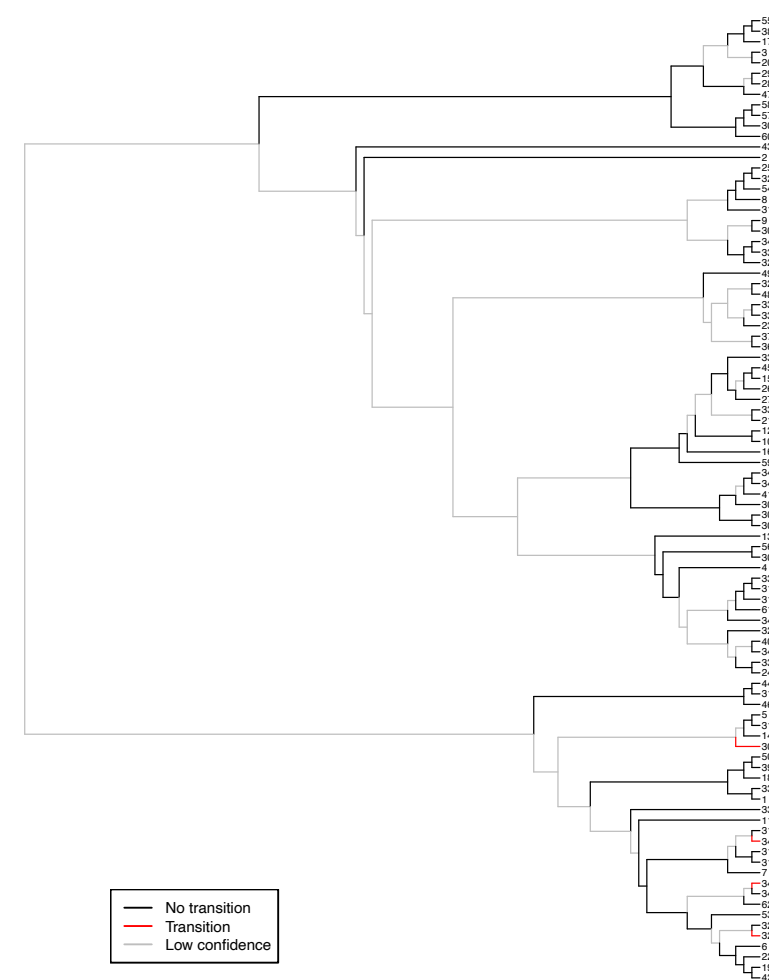

C)

GdpP premature STOP

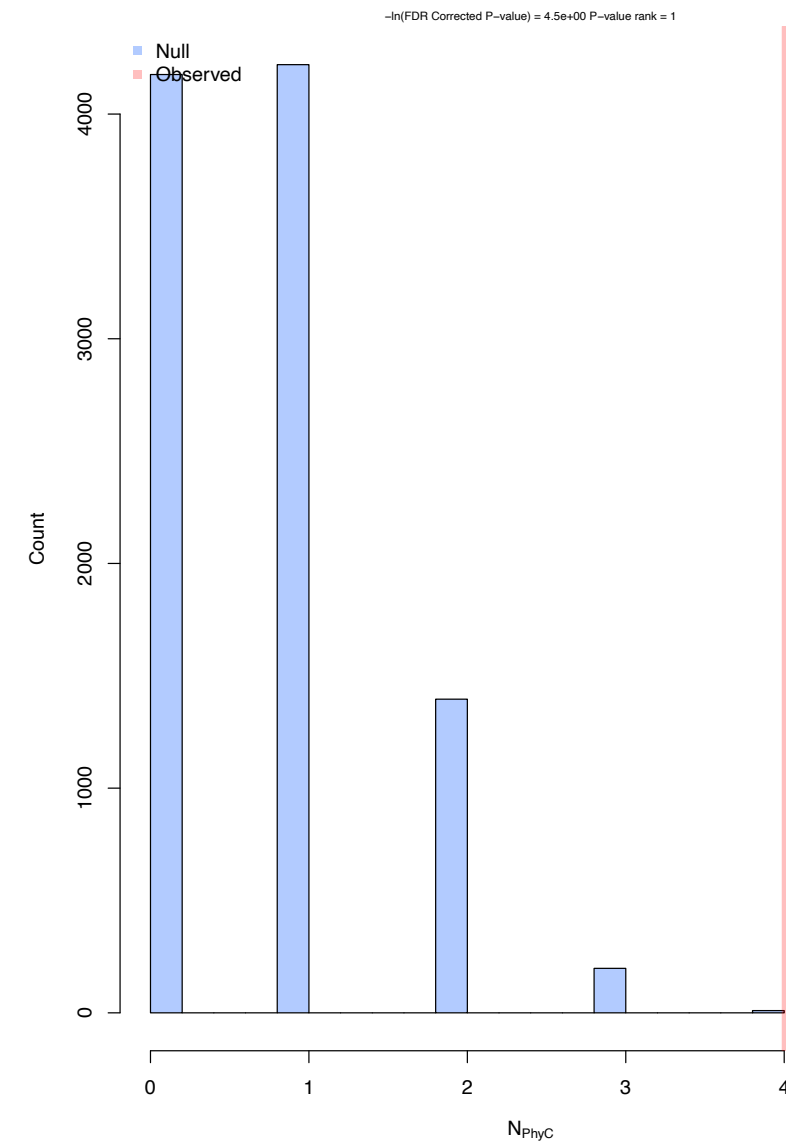

Supplement: FIG S6 [file mbio.03196-21-sf006.pdf]
