# Supplementary material for: Comparative Genomics of Borderline Oxacillin-Resistant Staphylococcus aureus Detected during a Pseudo-outbreak of Methicillin-Resistant S. aureus in a Neonatal Intensive Care Unit
Source: mBio. 2022 Jan 18;13(1):e03196-21. doi: 10.1128/mbio.03196-21 (PMC8764539; doi:10.1128/mbio.03196-21)
Supplement: TABLE S2 [file mbio.03196-21-st002.docx]

**Table S2: Assembly statistics for each sequenced isolate.**

| Assembly | Id | Completeness | Contamination | Strain heterogeneity | # contigs (>= 0 bp) | # contigs (>= 1000 bp) | # contigs (>= 5000 bp) | # contigs (>= 10000 bp) | # contigs (>= 25000 bp) | # contigs (>= 50000 bp) | Total length (>= 0 bp) | Total length (>= 1000 bp) | Total length (>= 5000 bp) | Total length (>= 10000 bp) | Total length (>= 25000 bp) | Total length (>= 50000 bp) | # contigs | Largest contig | Total length | GC (%) | N50 | N75 | L50 | L75 | # N's per 100 kbp |  |
| --- | --- | --- | --- | --- | --- | --- | --- | --- | --- | --- | --- | --- | --- | --- | --- | --- | --- | --- | --- | --- | --- | --- | --- | --- | --- | --- |
| 1 | g__Staphylococcus | 99.51 | 0.25 | 0 | 96 | 42 | 34 | 31 | 25 | 17 | 2,820,513 | 2,809,257 | 2,792,580 | 2,773,898 | 2,670,212 | 2,393,403 | 46 | 318,994 | 2,811,776 | 33 | 175,697 | 78,744 | 6 | 13 | 10.35 |  |
| 2 | g__Staphylococcus | 98.95 | 0.1 | 0 | 57 | 15 | 10 | 10 | 10 | 7 | 2,821,413 | 2,812,757 | 2,801,091 | 2,801,091 | 2,801,091 | 2,670,687 | 19 | 1,028,528 | 2,815,296 | 33 | 975,452 | 332,414 | 2 | 3 | 13.78 |  |
| 3 | g__Staphylococcus | 99.51 | 0.08 | 0 | 87 | 22 | 17 | 16 | 12 | 11 | 2,664,587 | 2,653,822 | 2,642,188 | 2,635,467 | 2,566,017 | 2,529,409 | 23 | 713,367 | 2,654,746 | 33 | 314,176 | 133,824 | 3 | 6 | 18.46 |  |
| 4 | g__Staphylococcus | 99.51 | 0.08 | 0 | 84 | 22 | 17 | 17 | 15 | 13 | 2,771,351 | 2,759,740 | 2,745,292 | 2,745,292 | 2,702,370 | 2,633,362 | 25 | 403,813 | 2,761,597 | 33 | 293,964 | 169,167 | 4 | 7 | 31.79 |  |
| 5 | g__Staphylococcus | 99.51 | 0.13 | 0 | 83 | 17 | 13 | 12 | 11 | 7 | 2,805,157 | 2,794,569 | 2,786,461 | 2,780,297 | 2,759,514 | 2,590,627 | 20 | 1,004,201 | 2,796,406 | 33 | 861,117 | 328,124 | 2 | 3 | 20.67 |  |
| 6 | g__Staphylococcus | 99.51 | 0.08 | 0 | 97 | 24 | 17 | 17 | 16 | 12 | 2,696,391 | 2,681,716 | 2,663,170 | 2,663,170 | 2,641,229 | 2,484,942 | 25 | 548,842 | 2,682,237 | 33 | 410,626 | 105,417 | 3 | 7 | 36.09 |  |
| 7 | g__Staphylococcus | 99.51 | 0.08 | 0 | 95 | 22 | 17 | 16 | 15 | 10 | 2,693,514 | 2,679,437 | 2,667,603 | 2,661,930 | 2,639,989 | 2,454,521 | 23 | 536,197 | 2,679,958 | 33 | 410,474 | 136,929 | 3 | 6 | 32.24 |  |
| 8 | g__Staphylococcus | 99.51 | 0.08 | 0 | 91 | 22 | 16 | 16 | 15 | 12 | 2,714,967 | 2,702,923 | 2,688,583 | 2,688,583 | 2,670,723 | 2,537,929 | 23 | 561,524 | 2,703,471 | 33 | 403,753 | 131,801 | 3 | 7 | 43.46 |  |
| 9 | g__Staphylococcus | 99.51 | 0.11 | 0 | 74 | 15 | 12 | 12 | 11 | 10 | 2,781,149 | 2,770,590 | 2,764,941 | 2,764,941 | 2,744,157 | 2,705,411 | 17 | 783,112 | 2,771,902 | 33 | 259,214 | 197,042 | 3 | 6 | 35.21 |  |
| 10 | g__Staphylococcus | 99.51 | 0.69 | 0 | 91 | 30 | 23 | 22 | 19 | 14 | 2,874,464 | 2,863,792 | 2,850,562 | 2,845,121 | 2,792,498 | 2,614,248 | 30 | 542,115 | 2,863,792 | 33 | 273,510 | 106,473 | 4 | 8 | 13.65 |  |
| 11 | g__Staphylococcus | 99.51 | 0.19 | 50 | 115 | 35 | 31 | 31 | 26 | 15 | 2,683,918 | 2,668,436 | 2,655,677 | 2,655,677 | 2,561,314 | 2,153,300 | 35 | 397,430 | 2,668,436 | 33 | 140,899 | 61,862 | 6 | 13 | 7.46 |  |
| 12 | g__Staphylococcus | 99.51 | 0.64 | 0 | 87 | 24 | 17 | 16 | 12 | 11 | 2,825,354 | 2,814,929 | 2,801,242 | 2,795,801 | 2,720,990 | 2,681,925 | 24 | 834,827 | 2,814,929 | 33 | 332,185 | 173,132 | 3 | 5 | 13.82 |  |
| 13 | g__Staphylococcus | 99.51 | 0.08 | 0 | 130 | 18 | 15 | 15 | 14 | 11 | 2,787,711 | 2,770,295 | 2,764,647 | 2,764,647 | 2,747,976 | 2,630,374 | 20 | 756,166 | 2,771,366 | 33 | 311,117 | 126,750 | 3 | 6 | 38.9 |  |
| 14 | g__Staphylococcus | 99.51 | 0.13 | 0 | 71 | 14 | 10 | 10 | 10 | 8 | 2,728,284 | 2,718,720 | 2,710,583 | 2,710,583 | 2,710,583 | 2,628,212 | 16 | 987,854 | 2,720,190 | 33 | 497,884 | 281,703 | 2 | 4 | 28.64 |  |
| 15 | g__Staphylococcus | 99.51 | 0.64 | 0 | 88 | 21 | 15 | 14 | 12 | 10 | 2,861,451 | 2,849,613 | 2,836,920 | 2,831,479 | 2,791,939 | 2,706,296 | 22 | 986,123 | 2,850,139 | 33 | 820,827 | 345,457 | 2 | 3 | 13.79 |  |
| 16 | g__Staphylococcus | 99.51 | 0.08 | 0 | 89 | 26 | 17 | 16 | 11 | 10 | 2,795,566 | 2,783,960 | 2,763,647 | 2,758,201 | 2,668,696 | 2,629,630 | 26 | 971,863 | 2,783,960 | 33 | 546,548 | 293,725 | 2 | 4 | 14.12 |  |
| 17 | g__Staphylococcus | 99.51 | 0.08 | 0 | 65 | 19 | 15 | 15 | 13 | 10 | 2,751,469 | 2,742,785 | 2,735,990 | 2,735,990 | 2,696,037 | 2,595,470 | 22 | 600,595 | 2,744,822 | 33 | 271,203 | 202,584 | 4 | 6 | 25.03 |  |
| 18 | g__Staphylococcus | 99.37 | 0.22 | 0 | 92 | 35 | 30 | 28 | 23 | 19 | 2,835,493 | 2,824,931 | 2,814,223 | 2,801,963 | 2,706,623 | 2,572,877 | 37 | 497,138 | 2,826,152 | 33 | 155,042 | 78,704 | 6 | 12 | 13.91 |  |
| 19 | g__Staphylococcus | 99.51 | 0.08 | 0 | 113 | 27 | 20 | 19 | 18 | 13 | 2,739,139 | 2,720,301 | 2,698,916 | 2,690,901 | 2,668,960 | 2,467,389 | 30 | 564,697 | 2,722,462 | 33 | 209,929 | 134,253 | 4 | 8 | 31.74 |  |
| 20 | g__Staphylococcus | 99.23 | 0.08 | 0 | 91 | 30 | 25 | 25 | 22 | 14 | 2,802,105 | 2,790,010 | 2,777,074 | 2,777,074 | 2,713,719 | 2,431,286 | 33 | 376,004 | 2,792,492 | 33 | 210,835 | 85,184 | 5 | 10 | 17.58 |  |
| 21 | g__Staphylococcus | 99.51 | 0.64 | 0 | 117 | 31 | 22 | 21 | 15 | 13 | 2,872,257 | 2,857,438 | 2,838,350 | 2,832,909 | 2,716,129 | 2,652,176 | 31 | 821,239 | 2,857,438 | 33 | 295,823 | 106,731 | 3 | 7 | 10.11 |  |
| 22 | g__Staphylococcus | 99.51 | 0.08 | 0 | 109 | 25 | 17 | 17 | 16 | 12 | 2,716,846 | 2,698,839 | 2,676,077 | 2,676,077 | 2,655,326 | 2,498,380 | 26 | 562,350 | 2,699,793 | 33 | 411,395 | 105,417 | 3 | 7 | 39.37 |  |
| 23 | g__Staphylococcus | 99.48 | 0.08 | 0 | 95 | 25 | 17 | 12 | 9 | 7 | 2,830,390 | 2,813,981 | 2,785,156 | 2,743,696 | 2,691,253 | 2,604,022 | 30 | 1,067,405 | 2,817,064 | 33 | 699,993 | 215,097 | 2 | 4 | 20.8 |  |
| 24 | g__Staphylococcus | 99.51 | 0.08 | 0 | 129 | 29 | 26 | 25 | 21 | 16 | 2,724,496 | 2,705,283 | 2,699,581 | 2,694,133 | 2,613,746 | 2,435,401 | 32 | 355,484 | 2,707,534 | 33 | 208,543 | 104,310 | 5 | 11 | 10.49 |  |
| 25 | g__Staphylococcus | 99.51 | 0.08 | 0 | 88 | 21 | 18 | 18 | 17 | 14 | 2,715,912 | 2,703,109 | 2,697,363 | 2,697,363 | 2,686,011 | 2,565,056 | 24 | 545,657 | 2,705,073 | 33 | 212,729 | 148,293 | 5 | 8 | 35.86 |  |
| 26 | g__Staphylococcus | 99.51 | 0.64 | 0 | 85 | 21 | 16 | 15 | 13 | 11 | 2,737,311 | 2,726,708 | 2,716,889 | 2,711,448 | 2,671,605 | 2,596,776 | 21 | 822,175 | 2,726,708 | 33 | 298,438 | 203,727 | 3 | 5 | 7 |  |
| 27 | g__Staphylococcus | 99.51 | 0.64 | 0 | 130 | 27 | 17 | 16 | 14 | 13 | 2,886,004 | 2,863,451 | 2,841,052 | 2,835,611 | 2,796,073 | 2,757,008 | 33 | 987,657 | 2,867,597 | 33 | 313,453 | 155,052 | 3 | 6 | 10.25 |  |
| 28 | g__Staphylococcus | 99.51 | 0.08 | 0 | 78 | 29 | 24 | 23 | 20 | 13 | 2,783,188 | 2,774,616 | 2,761,383 | 2,752,343 | 2,691,931 | 2,439,165 | 29 | 701,151 | 2,774,616 | 33 | 316,238 | 88,284 | 3 | 9 | 17.52 |  |
| 29 | g__Staphylococcus | 99.51 | 0.66 | 40 | 171 | 49 | 27 | 24 | 19 | 13 | 2,859,910 | 2,831,549 | 2,777,712 | 2,755,720 | 2,672,813 | 2,472,672 | 60 | 382,792 | 2,839,650 | 33 | 220,128 | 95,828 | 5 | 9 | 27.57 |  |
| 30 | g__Staphylococcus | 99.51 | 0.08 | 0 | 68 | 24 | 17 | 17 | 16 | 12 | 2,704,592 | 2,695,748 | 2,677,222 | 2,677,222 | 2,657,009 | 2,505,981 | 24 | 448,899 | 2,695,748 | 33 | 291,054 | 179,127 | 4 | 8 | 18.03 |  |
| 31 | g__Staphylococcus | 99.51 | 0.13 | 0 | 82 | 21 | 13 | 12 | 9 | 5 | 2,762,517 | 2,751,516 | 2,736,100 | 2,729,718 | 2,674,089 | 2,511,130 | 24 | 1,300,055 | 2,753,737 | 33 | 601,983 | 427,318 | 2 | 3 | 38.53 |  |
| 32 | g__Staphylococcus | 99.51 | 0.08 | 0 | 77 | 22 | 17 | 17 | 13 | 11 | 2,723,016 | 2,711,942 | 2,701,279 | 2,701,279 | 2,616,818 | 2,530,459 | 24 | 514,205 | 2,713,165 | 33 | 420,697 | 257,823 | 3 | 5 | 28.79 |  |
| 33 | g__Staphylococcus | 99.51 | 0.22 | 0 | 101 | 40 | 34 | 32 | 28 | 19 | 2,809,369 | 2,796,843 | 2,784,763 | 2,772,767 | 2,694,778 | 2,370,069 | 44 | 305,046 | 2,799,339 | 33 | 150,804 | 61,520 | 8 | 15 | 13.82 |  |
| 34 | g__Staphylococcus | 99.51 | 0.11 | 0 | 113 | 23 | 21 | 21 | 19 | 13 | 2,732,108 | 2,714,176 | 2,709,970 | 2,709,970 | 2,664,763 | 2,444,329 | 28 | 353,579 | 2,717,258 | 33 | 255,954 | 140,948 | 5 | 8 | 25.06 |  |
| 36 | g__Staphylococcus | 99.51 | 0.1 | 0 | 117 | 27 | 20 | 20 | 19 | 14 | 2,748,561 | 2,732,785 | 2,714,166 | 2,714,166 | 2,697,925 | 2,531,198 | 27 | 589,810 | 2,732,785 | 33 | 382,857 | 84,371 | 3 | 7 | 21.59 |  |
| 37 | g__Staphylococcus | 99.51 | 0.1 | 0 | 118 | 23 | 16 | 15 | 13 | 10 | 2,822,223 | 2,803,709 | 2,790,325 | 2,782,130 | 2,745,950 | 2,627,243 | 27 | 638,211 | 2,806,135 | 33 | 591,289 | 159,366 | 3 | 5 | 20.7 |  |
| 38 | g__Staphylococcus | 99.51 | 0.08 | 0 | 86 | 24 | 17 | 17 | 15 | 11 | 2,690,963 | 2,678,329 | 2,665,270 | 2,665,270 | 2,625,291 | 2,498,404 | 28 | 536,844 | 2,681,076 | 33 | 296,685 | 129,753 | 4 | 7 | 7.12 |  |
| 39 | g__Staphylococcus | 99.51 | 0.22 | 0 | 91 | 34 | 29 | 28 | 23 | 16 | 2,803,864 | 2,792,974 | 2,781,455 | 2,776,246 | 2,680,942 | 2,426,665 | 36 | 484,733 | 2,794,191 | 33 | 174,963 | 67,681 | 5 | 11 | 10.52 |  |
| 40 | g__Staphylococcus | 99.51 | 0.08 | 0 | 92 | 21 | 19 | 19 | 17 | 15 | 2,700,708 | 2,687,758 | 2,683,480 | 2,683,480 | 2,642,878 | 2,573,388 | 24 | 570,432 | 2,689,813 | 33 | 250,364 | 118,569 | 4 | 8 | 21.93 |  |
| 41 | g__Staphylococcus | 99.51 | 0.08 | 0 | 79 | 21 | 15 | 15 | 14 | 12 | 2,719,537 | 2,709,035 | 2,692,330 | 2,692,330 | 2,675,360 | 2,587,439 | 22 | 795,063 | 2,709,583 | 33 | 323,776 | 151,587 | 3 | 7 | 17.94 |  |
| 42 | g__Staphylococcus | 99.51 | 0.08 | 0 | 134 | 29 | 22 | 21 | 19 | 14 | 2,742,662 | 2,718,282 | 2,696,899 | 2,687,873 | 2,641,847 | 2,440,885 | 33 | 463,193 | 2,721,114 | 33 | 158,710 | 100,885 | 4 | 9 | 35.83 |  |
| 43 | g__Staphylococcus | 99.4 | 0.13 | 0 | 95 | 17 | 15 | 15 | 12 | 10 | 2,731,883 | 2,719,010 | 2,714,344 | 2,714,344 | 2,660,343 | 2,578,284 | 17 | 623,326 | 2,719,010 | 33 | 385,583 | 199,021 | 3 | 5 | 21.7 |  |
| 44 | g__Staphylococcus | 99.51 | 0.22 | 0 | 108 | 36 | 30 | 28 | 22 | 16 | 2,707,166 | 2,693,347 | 2,677,063 | 2,661,758 | 2,540,715 | 2,318,923 | 37 | 303,985 | 2,694,056 | 33 | 135,743 | 98,225 | 7 | 12 | 32.85 |  |
| 45 | g__Staphylococcus | 99.51 | 0.64 | 0 | 116 | 29 | 21 | 18 | 13 | 11 | 2,902,784 | 2,886,976 | 2,867,346 | 2,845,261 | 2,741,522 | 2,672,381 | 31 | 956,930 | 2,888,059 | 33 | 547,381 | 109,381 | 2 | 5 | 6.72 |  |
| 46 | g__Staphylococcus | 98.95 | 0.25 | 0 | 129 | 41 | 32 | 28 | 22 | 16 | 2,820,520 | 2,801,462 | 2,782,065 | 2,753,353 | 2,633,567 | 2,386,204 | 46 | 380,086 | 2,805,602 | 33 | 169,714 | 76,933 | 6 | 12 | 27.41 |  |
| 47 | g__Staphylococcus | 99.51 | 0.08 | 0 | 63 | 27 | 21 | 21 | 18 | 14 | 2,771,254 | 2,764,961 | 2,749,455 | 2,749,455 | 2,688,238 | 2,544,231 | 27 | 471,603 | 2,764,961 | 33 | 187,299 | 120,462 | 5 | 9 | 21.16 |  |
| 48 | g__Staphylococcus | 99.45 | 0.08 | 0 | 54 | 14 | 11 | 10 | 9 | 8 | 2,763,241 | 2,756,642 | 2,750,125 | 2,743,444 | 2,722,738 | 2,683,797 | 15 | 805,942 | 2,757,238 | 33 | 728,605 | 229,130 | 2 | 4 | 21.18 |  |
| 49 | g__Staphylococcus | 99.51 | 0.08 | 0 | 55 | 12 | 8 | 8 | 8 | 6 | 2,707,839 | 2,699,319 | 2,688,038 | 2,688,038 | 2,688,038 | 2,614,999 | 12 | 1,342,337 | 2,699,319 | 33 | 358,605 | 328,586 | 2 | 3 | 18.04 |  |
| 50 | g__Staphylococcus | 99.51 | 0.22 | 0 | 87 | 36 | 27 | 25 | 22 | 17 | 2,858,026 | 2,847,841 | 2,827,021 | 2,815,024 | 2,752,398 | 2,565,136 | 39 | 406,726 | 2,849,670 | 33 | 169,490 | 85,069 | 6 | 11 | 6.91 |  |
| 53 | g__Staphylococcus | 99.51 | 0.08 | 0 | 99 | 24 | 16 | 16 | 15 | 11 | 2,711,550 | 2,696,012 | 2,673,189 | 2,673,189 | 2,651,248 | 2,494,395 | 25 | 564,656 | 2,696,533 | 33 | 410,568 | 131,753 | 3 | 6 | 32.41 |  |
| 54 | g__Staphylococcus | 99.51 | 0.08 | 0 | 65 | 15 | 12 | 12 | 12 | 9 | 2,702,325 | 2,693,119 | 2,687,933 | 2,687,933 | 2,687,933 | 2,555,298 | 17 | 967,666 | 2,694,348 | 33 | 448,082 | 257,912 | 2 | 4 | 32.66 |  |
| 55 | g__Staphylococcus | 99.51 | 0.08 | 0 | 87 | 27 | 18 | 18 | 16 | 12 | 2,753,367 | 2,743,359 | 2,724,051 | 2,724,051 | 2,684,092 | 2,553,897 | 29 | 455,136 | 2,744,755 | 33 | 310,721 | 129,766 | 4 | 8 | 10.42 |  |
| 56 | g__Staphylococcus | 99.41 | 0.08 | 0 | 93 | 17 | 14 | 14 | 12 | 11 | 2,770,210 | 2,757,369 | 2,750,140 | 2,750,140 | 2,711,757 | 2,673,732 | 17 | 570,962 | 2,757,369 | 33 | 429,058 | 158,147 | 3 | 6 | 24.26 |  |
| 57 | g__Staphylococcus | 99.51 | 0.08 | 0 | 69 | 22 | 16 | 16 | 15 | 12 | 2,716,338 | 2,709,032 | 2,694,738 | 2,694,738 | 2,674,523 | 2,574,743 | 22 | 696,170 | 2,709,032 | 33 | 232,825 | 167,248 | 4 | 7 | 17.9 |  |
| 58 | g__Staphylococcus | 99.51 | 0.08 | 0 | 62 | 21 | 16 | 16 | 14 | 12 | 2,709,609 | 2,702,779 | 2,689,941 | 2,689,941 | 2,649,990 | 2,580,573 | 21 | 472,413 | 2,702,779 | 33 | 244,104 | 187,573 | 4 | 7 | 21.72 |  |
| 59 | g__Staphylococcus | 99.51 | 0.11 | 0 | 124 | 33 | 29 | 26 | 20 | 14 | 2,833,613 | 2,817,829 | 2,809,392 | 2,787,625 | 2,680,377 | 2,446,387 | 34 | 417,998 | 2,818,432 | 33 | 210,480 | 111,046 | 5 | 10 | 24.09 |  |
| 60 | g__Staphylococcus | 99.51 | 0.08 | 0 | 90 | 32 | 24 | 23 | 19 | 14 | 2,776,637 | 2,767,595 | 2,745,508 | 2,739,487 | 2,666,166 | 2,485,883 | 32 | 379,880 | 2,767,595 | 33 | 197,331 | 107,402 | 5 | 9 | 28.36 |  |
| 61 | g__Staphylococcus | 99.51 | 0.08 | 0 | 91 | 23 | 21 | 20 | 16 | 14 | 2,675,702 | 2,662,746 | 2,658,033 | 2,648,036 | 2,570,704 | 2,501,928 | 25 | 354,306 | 2,663,958 | 33 | 249,996 | 125,726 | 5 | 9 | 18.32 |  |
| 62 | g__Staphylococcus | 99.51 | 0.08 | 0 | 107 | 25 | 19 | 19 | 15 | 11 | 2,718,961 | 2,702,075 | 2,683,585 | 2,683,585 | 2,608,560 | 2,451,937 | 28 | 562,972 | 2,704,235 | 33 | 408,764 | 116,684 | 3 | 6 | 25.29 |  |
| 301 | g__Staphylococcus | 99.45 | 0.11 | 0 | 93 | 23 | 18 | 18 | 17 | 11 | 2,753,837 | 2,740,897 | 2,730,261 | 2,730,261 | 2,716,098 | 2,494,082 | 27 | 324,859 | 2,743,547 | 33 | 262,075 | 196,922 | 5 | 8 | 60.4 |  |
| 302 | g__Staphylococcus | 99.51 | 0.08 | 0 | 63 | 16 | 13 | 13 | 11 | 9 | 2,739,202 | 2,731,642 | 2,725,454 | 2,725,454 | 2,688,003 | 2,600,025 | 16 | 654,378 | 2,731,642 | 33 | 386,964 | 225,176 | 3 | 5 | 17.68 |  |
| 303 | g__Staphylococcus | 99.51 | 0.64 | 0 | 108 | 25 | 18 | 17 | 14 | 11 | 2,826,507 | 2,812,789 | 2,796,198 | 2,790,757 | 2,732,646 | 2,617,955 | 25 | 835,012 | 2,812,789 | 33 | 588,887 | 172,816 | 2 | 5 | 27.66 |  |
| 304 | g__Staphylococcus | 99.41 | 0.11 | 0 | 97 | 18 | 15 | 15 | 13 | 11 | 2,772,368 | 2,760,376 | 2,753,155 | 2,753,155 | 2,714,775 | 2,647,101 | 18 | 437,288 | 2,760,376 | 33 | 388,306 | 182,830 | 4 | 6 | 31.92 |  |
| 305 | g__Staphylococcus | 99.45 | 0.13 | 0 | 60 | 14 | 9 | 9 | 8 | 5 | 2,698,288 | 2,690,739 | 2,682,070 | 2,682,070 | 2,661,364 | 2,533,808 | 16 | 1,278,060 | 2,692,018 | 33 | 902,921 | 902,921 | 2 | 2 | 21.81 |  |
| 306 | g__Staphylococcus | 99.51 | 0.08 | 0 | 76 | 16 | 13 | 13 | 12 | 11 | 2,738,998 | 2,728,332 | 2,723,018 | 2,723,018 | 2,701,102 | 2,662,130 | 17 | 792,787 | 2,728,880 | 33 | 286,563 | 150,086 | 3 | 6 | 14.36 |  |
| 307 | g__Staphylococcus | 99.51 | 0.08 | 0 | 74 | 18 | 15 | 15 | 13 | 11 | 2,737,909 | 2,727,855 | 2,722,315 | 2,722,315 | 2,683,428 | 2,595,417 | 20 | 792,787 | 2,729,170 | 33 | 286,679 | 151,552 | 3 | 6 | 10.7 |  |
| 308 | g__Staphylococcus | 99.32 | 0.08 | 0 | 57 | 21 | 17 | 16 | 13 | 10 | 2,784,347 | 2,778,168 | 2,769,312 | 2,762,356 | 2,721,809 | 2,602,526 | 21 | 1,063,139 | 2,778,168 | 33 | 382,810 | 207,172 | 2 | 5 | 31.1 |  |
| 309 | g__Staphylococcus | 99.51 | 0.1 | 0 | 83 | 25 | 18 | 16 | 14 | 11 | 2,803,826 | 2,794,221 | 2,774,108 | 2,757,382 | 2,717,483 | 2,625,105 | 26 | 385,786 | 2,795,011 | 33 | 316,103 | 202,040 | 4 | 7 | 24.4 |  |
| 310 | g__Staphylococcus | 99.51 | 0.08 | 0 | 111 | 31 | 24 | 21 | 18 | 14 | 2,775,528 | 2,759,681 | 2,741,629 | 2,720,475 | 2,660,436 | 2,503,015 | 34 | 562,655 | 2,761,463 | 33 | 236,285 | 117,778 | 4 | 9 | 31.43 |  |
| 311 | g__Staphylococcus | 99.51 | 0.08 | 0 | 143 | 39 | 31 | 26 | 19 | 14 | 2,818,019 | 2,793,769 | 2,773,943 | 2,739,743 | 2,621,853 | 2,433,790 | 47 | 562,655 | 2,799,508 | 33 | 197,159 | 92,436 | 5 | 10 | 27.36 |  |
| 312 | g__Staphylococcus | 99.51 | 0.08 | 0 | 112 | 32 | 25 | 22 | 18 | 14 | 2,775,559 | 2,759,660 | 2,741,612 | 2,720,457 | 2,641,827 | 2,484,358 | 35 | 562,655 | 2,761,442 | 33 | 236,285 | 118,249 | 4 | 9 | 28.21 |  |
| 314 | g__Staphylococcus | 99.51 | 0.22 | 0 | 80 | 28 | 25 | 24 | 20 | 14 | 2,728,786 | 2,718,981 | 2,712,215 | 2,705,427 | 2,632,563 | 2,411,552 | 29 | 389,396 | 2,719,976 | 33 | 170,008 | 98,229 | 5 | 10 | 32.1 |  |
| 315 | g__Staphylococcus | 99.51 | 0.08 | 0 | 54 | 14 | 11 | 11 | 11 | 8 | 2,706,207 | 2,699,043 | 2,692,724 | 2,692,724 | 2,692,724 | 2,559,929 | 16 | 760,417 | 2,700,194 | 33 | 501,762 | 354,227 | 3 | 4 | 36.07 |  |
| 316 | g__Staphylococcus | 99.51 | 0.08 | 0 | 128 | 31 | 25 | 23 | 17 | 14 | 2,714,419 | 2,694,309 | 2,677,065 | 2,663,231 | 2,552,050 | 2,442,455 | 35 | 436,681 | 2,696,966 | 33 | 215,835 | 110,841 | 5 | 9 | 21.69 |  |
| 318 | g__Staphylococcus | 99.51 | 0.08 | 0 | 127 | 30 | 24 | 22 | 17 | 14 | 2,713,792 | 2,691,017 | 2,673,893 | 2,660,978 | 2,565,980 | 2,457,145 | 37 | 436,681 | 2,696,283 | 33 | 215,835 | 110,841 | 5 | 9 | 21.81 |  |
| 320 | g__Staphylococcus | 99.51 | 0.1 | 0 | 88 | 23 | 17 | 15 | 13 | 10 | 2,760,697 | 2,750,502 | 2,734,357 | 2,717,631 | 2,677,732 | 2,584,820 | 24 | 765,770 | 2,751,292 | 33 | 316,102 | 202,040 | 3 | 6 | 28.46 |  |
| 321 | g__Staphylococcus | 99.51 | 0.08 | 0 | 122 | 24 | 18 | 18 | 17 | 14 | 2,753,195 | 2,732,563 | 2,716,174 | 2,716,174 | 2,694,233 | 2,567,439 | 27 | 560,554 | 2,734,724 | 33 | 410,626 | 134,105 | 3 | 7 | 42.71 |  |
| 322 | g__Staphylococcus | 99.51 | 0.08 | 0 | 89 | 23 | 19 | 18 | 16 | 12 | 2,756,247 | 2,742,763 | 2,734,153 | 2,725,625 | 2,697,385 | 2,556,134 | 26 | 469,413 | 2,744,806 | 33 | 263,568 | 151,219 | 5 | 8 | 14.14 |  |
| 323 | g__Staphylococcus | 99.51 | 0.13 | 0 | 68 | 29 | 21 | 18 | 16 | 14 | 2,777,285 | 2,769,807 | 2,747,992 | 2,722,933 | 2,693,048 | 2,626,518 | 31 | 383,281 | 2,771,397 | 33 | 200,699 | 131,233 | 5 | 9 | 24.07 |  |
| 324 | g__Staphylococcus | 99.23 | 0.64 | 0 | 93 | 21 | 16 | 15 | 12 | 10 | 2,866,894 | 2,854,707 | 2,845,216 | 2,839,775 | 2,775,983 | 2,690,360 | 22 | 980,743 | 2,855,233 | 33 | 834,636 | 345,459 | 2 | 3 | 16.95 |  |
| 325 | g__Staphylococcus | 99.41 | 0.08 | 0 | 108 | 24 | 22 | 21 | 17 | 14 | 2,799,833 | 2,784,296 | 2,779,597 | 2,773,445 | 2,697,145 | 2,597,895 | 26 | 333,918 | 2,785,978 | 33 | 256,509 | 170,214 | 5 | 8 | 20.93 |  |
| 327 | g__Staphylococcus | 99.51 | 0.08 | 0 | 71 | 18 | 15 | 15 | 14 | 13 | 2,703,734 | 2,694,783 | 2,686,217 | 2,686,217 | 2,669,509 | 2,630,787 | 18 | 599,396 | 2,694,783 | 33 | 284,797 | 159,882 | 4 | 7 | 14.47 |  |
| 328 | g__Staphylococcus | 99.42 | 0.08 | 0 | 111 | 22 | 16 | 16 | 15 | 11 | 2,693,209 | 2,674,011 | 2,657,622 | 2,657,622 | 2,635,681 | 2,478,657 | 25 | 560,550 | 2,676,172 | 33 | 410,628 | 134,528 | 3 | 7 | 40.17 |  |
| 329 | g__Staphylococcus | 99.4 | 0.27 | 0 | 117 | 60 | 44 | 36 | 27 | 18 | 2,899,619 | 2,886,868 | 2,849,059 | 2,798,047 | 2,661,842 | 2,325,095 | 66 | 417,935 | 2,890,944 | 33 | 108,969 | 60,679 | 7 | 16 | 10 |  |
| 330 | g__Staphylococcus | 99.51 | 0.64 | 0 | 112 | 36 | 27 | 24 | 19 | 15 | 2,912,533 | 2,897,734 | 2,878,139 | 2,853,426 | 2,771,275 | 2,610,163 | 39 | 426,040 | 2,899,439 | 33 | 238,406 | 105,779 | 5 | 9 | 23.35 |  |
| 331 | g__Staphylococcus | 99.51 | 0.08 | 0 | 87 | 21 | 19 | 19 | 15 | 12 | 2,745,539 | 2,735,315 | 2,731,091 | 2,731,091 | 2,655,553 | 2,538,285 | 22 | 570,246 | 2,735,906 | 33 | 249,432 | 190,791 | 4 | 7 | 17.84 |  |
| 332 | g__Staphylococcus | 99.48 | 0.08 | 0 | 64 | 17 | 11 | 9 | 8 | 6 | 2,772,470 | 2,763,667 | 2,745,932 | 2,730,242 | 2,709,586 | 2,621,444 | 19 | 1,368,543 | 2,764,855 | 33 | 701,096 | 169,878 | 2 | 3 | 17.32 |  |
| 333 | g__Staphylococcus | 99.46 | 0.64 | 0 | 100 | 30 | 22 | 20 | 15 | 11 | 2,862,422 | 2,849,735 | 2,831,882 | 2,818,274 | 2,714,618 | 2,572,844 | 30 | 860,895 | 2,849,735 | 33 | 657,756 | 106,414 | 2 | 6 | 10.25 |  |
| 334 | g__Staphylococcus | 99.51 | 0.08 | 0 | 124 | 30 | 24 | 22 | 16 | 14 | 2,759,149 | 2,738,219 | 2,721,589 | 2,708,482 | 2,597,274 | 2,528,438 | 36 | 482,740 | 2,742,445 | 33 | 215,835 | 110,996 | 5 | 9 | 17.61 |  |
| 335 | g__Staphylococcus | 99.51 | 0.08 | 0 | 54 | 26 | 22 | 20 | 16 | 15 | 2,769,252 | 2,764,895 | 2,756,015 | 2,740,277 | 2,668,135 | 2,629,401 | 26 | 322,032 | 2,764,895 | 33 | 247,054 | 125,652 | 5 | 9 | 20.76 |  |
| 336 | g__Staphylococcus | 99.51 | 0.64 | 0 | 92 | 24 | 17 | 16 | 13 | 9 | 2,854,658 | 2,842,210 | 2,825,533 | 2,820,092 | 2,761,676 | 2,602,313 | 24 | 878,154 | 2,842,210 | 33 | 657,606 | 295,835 | 2 | 4 | 10.2 |  |
| 337 | g__Staphylococcus | 99.51 | 0.11 | 0 | 86 | 15 | 13 | 13 | 12 | 10 | 2,765,974 | 2,753,131 | 2,748,363 | 2,748,363 | 2,731,655 | 2,647,724 | 16 | 1,306,960 | 2,753,907 | 33 | 325,151 | 170,745 | 2 | 4 | 17.83 |  |
| 338 | g__Staphylococcus | 99.48 | 0.08 | 0 | 87 | 28 | 22 | 20 | 19 | 14 | 2,772,852 | 2,761,633 | 2,743,909 | 2,728,246 | 2,707,577 | 2,502,587 | 31 | 540,669 | 2,763,637 | 33 | 222,575 | 108,432 | 4 | 9 | 17.51 |  |
| 339 | g__Staphylococcus | 99.51 | 0.08 | 0 | 95 | 20 | 15 | 15 | 14 | 10 | 2,729,888 | 2,715,845 | 2,704,219 | 2,704,219 | 2,682,278 | 2,525,655 | 21 | 584,480 | 2,716,553 | 33 | 456,298 | 136,983 | 3 | 5 | 17.56 |  |
| 340 | g__Staphylococcus | 99.51 | 0.11 | 0 | 81 | 13 | 11 | 11 | 10 | 9 | 2,766,532 | 2,754,593 | 2,749,825 | 2,749,825 | 2,733,117 | 2,694,310 | 13 | 1,307,888 | 2,754,593 | 33 | 616,827 | 170,584 | 2 | 3 | 17.75 |  |
| 341 | g__Staphylococcus | 99.51 | 0.08 | 0 | 72 | 18 | 15 | 14 | 12 | 10 | 2,737,819 | 2,729,049 | 2,722,943 | 2,716,069 | 2,677,990 | 2,590,012 | 18 | 795,635 | 2,729,049 | 33 | 396,720 | 167,208 | 3 | 6 | 7.26 |  |
| 342 | g__Staphylococcus | 99.51 | 0.08 | 0 | 80 | 20 | 17 | 16 | 14 | 12 | 2,739,254 | 2,729,273 | 2,723,184 | 2,716,310 | 2,678,859 | 2,590,942 | 20 | 795,635 | 2,729,273 | 33 | 229,732 | 151,588 | 3 | 7 | 7.14 |  |
| 343 | g__Staphylococcus | 99.51 | 0.08 | 0 | 109 | 25 | 18 | 18 | 16 | 12 | 2,693,927 | 2,675,464 | 2,655,156 | 2,655,156 | 2,618,238 | 2,460,660 | 28 | 549,371 | 2,677,669 | 33 | 410,570 | 107,093 | 3 | 7 | 32.53 |  |
| 344 | g__Staphylococcus | 99.51 | 0.08 | 0 | 105 | 23 | 17 | 17 | 15 | 11 | 2,693,898 | 2,675,271 | 2,656,871 | 2,656,871 | 2,618,924 | 2,461,298 | 26 | 557,137 | 2,677,476 | 33 | 410,570 | 118,845 | 3 | 6 | 28.76 |  |
| 345 | g__Staphylococcus | 99.51 | 0.11 | 0 | 81 | 18 | 16 | 16 | 13 | 10 | 2,720,695 | 2,709,944 | 2,705,245 | 2,705,245 | 2,644,050 | 2,537,614 | 19 | 570,986 | 2,710,451 | 33 | 399,244 | 249,935 | 3 | 6 | 17.75 |  |
| 346 | g__Staphylococcus | 99.51 | 0.15 | 0 | 127 | 31 | 24 | 21 | 18 | 14 | 2,778,454 | 2,758,754 | 2,740,699 | 2,719,544 | 2,659,505 | 2,502,710 | 34 | 562,655 | 2,760,536 | 33 | 236,285 | 117,778 | 4 | 9 | 24.67 |  |
| --------------------------------------------------------------------------------------------------------------------------------------------------------------------------------------------------------------------------------------------------------------------------------------------------------------------------------------------------------------------------------------------------------------------------------------------------------------------------------------------------------------------------------------------------------------------------------------------------------------------------------------- | | | | | | | | | | | | | | | | | | | | | | | | | | |
