# Supplementary material for: Comparative Genomics of Borderline Oxacillin-Resistant Staphylococcus aureus Detected during a Pseudo-outbreak of Methicillin-Resistant S. aureus in a Neonatal Intensive Care Unit
Source: mBio. 2022 Jan 18;13(1):e03196-21. doi: 10.1128/mbio.03196-21 (PMC8764539; doi:10.1128/mbio.03196-21)
Supplement: TABLE S3 [file mbio.03196-21-st003.docx]

**Table S3: List of amino acid (AA) substitutions by BORSA isolate.** AA substitutions in the transglycosylase and transpeptidase domain of the PBP proteins are in italics and bold, respectively. Stop codons and deletions are colored red.

| MLST type | BORSA isolate | PBP1 | PBP2 | PBP3 | PBP4 | GdpP |
| --- | --- | --- | --- | --- | --- | --- |
| **CC1** | 23 | D118N, V617M | - | G167R | T189S | I52V |
|  | 37 | K67R, D118N | - | - | T189S | T104I, Y168F |
|  | 323 | D118N, V617M | - | G167R | T189S | I52V |
|  | 332 | D118N, V617M | - | G167R | T189S | I52V |
|  | 338 | D118N, V617M | - | G167R | T189S | I52V |
| **ST5** | 20 | - | A285P, **A516S** | - | A25T, T189S | D105N, P392S |
|  | 29 | - | A285P, S707I | - | A25T, T189S | D105N, P392S |
| **ST15** | 34 | D118N | - | **S438T** | T189S | - |
|  | 316 | D118N | - | **S438T** | T189S | - |
|  | 318 | D118N | - | **S438T** | T189S | - |
|  | 334 | D118N | - | **S438T** | T189S | - |
|  | 345 | D118N | - | **S438T** | T189S | S196W, H558Y |
| **ST45** | 305 | **R353K**, **D480E**, S664T | G17S, E269Q, A285P | S225A, **M376V**, **D599E** | Y208F, V381F, E398A, R430I | E97STOP, I456V |
| **ST97** | 302 | D118N | A315E, **S576A** | **S438T** | C12F, T101R, H214C, E398A | - |
|  | 306 | D118N | A315E, **S576A** | **S438T** | C12F, T101R, H214C, E398A | - |
|  | 307 | D118N | A315E, **S576A** | **S438T** | C12F, T101R, H214C, E398A | - |
|  | 341 | D118N | A315E, **S576A** | **S438T** | C12F, T101R, H214C, E398A | - |
|  | 342 | D118N | A315E, **S576A** | **S438T** | C12F, T101R, H214C, E398A | - |
| **ST7** | 301 | - | A285P, **Q629P** | G167R | C12F, T101R, T189S, E398A | D349Y |
| **ST8** | 10 | D118N | A285P | V30I, **T385A**, **S438T**, D683N | A25T, A409T | - |
|  | 12 | D118N | A285P | V30I, **S438T**, D683N | A25T, A409T | - |
|  | 26 | D118N | A285P | V30I, **S438T**, D683N | A25T, A409T | - |
|  | 336 | D118N | A285P | V30I, **S438T**, D683N | A25T, A409T | - |
| **ST27** | 49 | - | *K209R*, A285P | **S438T** | A25T, T189S | I52V |
| **ST72** | 315 | - | A285P | K138Q | T189S, Q383K | - |
| **ST88** | 327 | D118N, T146A | A285P, A315E, **R379L** | **S386F** | V202E, P253T, E398A | - |
|  | 337 | D118N, T146A, **K487N** | A285P, A315E | **S386F** | V202E, P253T, E398A | - |
|  | 340 | D118N, T146A, **K487N** | A285P, A315E | **S386F** | V202E, P253T, E398A | - |
| **ST398** | 321 | **F465L**, **D480E**, D662N, S664T | D270E, **T439V**, **D489E**, T691A | **R504K**, **E563D**, D684N | E398A | I52V, Y228STOP |
|  | 328 | **F465L**, **D480E**, D662N, S664T | D270E, **T439V**, **D489E**, T691A | **R504K**, **E563D**, D684N | E398A | I52V |
|  | 339 | P121S, **F465L**, **D480E**, D662N, S664T | D270E, **T439V**, **D489E**, T691A | **R504K**, **E563D**, D684N | E398A | I52V |
|  | 344 | **F465L**, **D480E**, D662N, S664T | D270E, **T439V**, **D489E**, T691A | **R504K**, **E563D**, D684N | E398A | 33 bp deletion at nt 350 |
|  | 346 | **F465L**, **D480E**, D662N, S664T | D270E, **T439V**, **D489E**, T691A | **R504K**, **E563D**, D684N | E398A | Q126STOP |
