# Supplementary material for: Comparative Genomics of Borderline Oxacillin-Resistant Staphylococcus aureus Detected during a Pseudo-outbreak of Methicillin-Resistant S. aureus in a Neonatal Intensive Care Unit
Source: mBio. 2022 Jan 18;13(1):e03196-21. doi: 10.1128/mbio.03196-21 (PMC8764539; doi:10.1128/mbio.03196-21)
Supplement: TABLE S4 [file mbio.03196-21-st004.docx]

**Table S4: Bivariate association of the top Random Forest Classifier features with BORSA and MSSA status.** Significance determined via Fisher’s Exact test with False Discovery Rate (FDR) correction. Feature correlation and anti-correlation with BORSA phenotype is noted.

| Feature | Present in BORSA | Absent in BORSA | Present in MSSA | Absent in MSSA | Frequency in BORSA | Frequency in MSSA | p-value | FDR-corrected p-value | BORSA correlation |
| --- | --- | --- | --- | --- | --- | --- | --- | --- | --- |
| A285P | 12 | 49 | 27 | 32 | 19.7% | 45.8% | 3.27E-03 | 5.45E-03 | - |
| PBP2 End Truncation | 0 | 61 | 6 | 53 | 0.0% | 10.2% | 1.23E-02 | 1.23E-02 | - |
| I52V | 7 | 54 | 24 | 35 | 11.5% | 40.7% | 3.25E-04 | 8.13E-04 | - |
| GdpP Premature STOP | 16 | 45 | 0 | 59 | 26.2% | 0.0% | 1.01E-05 | 5.03E-05 | + |
| T189S | 14 | 47 | 28 | 31 | 23.0% | 47.5% | 7.11E-03 | 8.89E-03 | - |
